# Functional and regulatory diversity of homeobox-leucine zipper transcription factors *BnaHB6* under dehydration and salt stress in *Brassica napus* L.

**DOI:** 10.1007/s11103-024-01465-6

**Published:** 2024-05-15

**Authors:** Natalia Żyła, Agata Cieśla, Laurencja Szała, Danuta Babula-Skowrońska

**Affiliations:** 1 Institute of Plant Genetics, Polish Academy of Sciences, Strzeszyńska 34, 60-479 Poznań, Poland; 2 Laboratory of Biotechnology, Faculty of Biology, Institute of Molecular Biology and Biotechnology, Adam Mickiewicz University in Poznań, Poznań, Poland; 3 Department of Oilseed Crops, Poznań Division, Plant Breeding and Acclimatization Institute-National Research Institute in Radzików, Strzeszyńska 36, 60‑479 Poznań, Poland

**Keywords:** HD-Zip I subfamily, *BnaHB6* homologues, Abiotic stress, Circadian rhythm, Functional divergence, *Brassica napus*

## Abstract

The plant-specific homeodomain-leucine zipper I subfamily is involved in the regulation of various biological processes, particularly growth, development and stress response. In the present study, we characterized four *BnaHB6* homologues from *Brassica napus*. All BnaHB6 proteins have transcriptional activation activity. Structural and functional data indicate the complex role of *BnaHB6* genes in regulating biological processes, with some functions conserved and others diverged. Transcriptional analyzes revealed that they are induced in a similar manner in different tissues but show different expression patterns in response to stress and circadian rhythm. Only the *BnaA09HB6* and *BnaC08HB6* genes are expressed under dehydration and salt stress, and in darkness. The partial transcriptional overlap of *BnaHB6*s with the evolutionarily related genes *BnaHB5* and *BnaHB16* was also observed. Transgenic *Arabidopsis thaliana* plants expressing a single pro*BnaHB6*::GUS partially confirmed the expression results. Bioinformatic analysis allowed the identification of TF-binding sites in the *BnaHB6* promoters that may control their expression under stress and circadian rhythm. ChIP-qPCR analysis revealed that *BnaA09HB6* and *BnaC08HB6* bind directly to the promoters of the target genes *BnaABF4* and *BnaDREB2A*. Comparison of their expression patterns in the WT plants and the *bnac08hb6* mutant showed that *BnaC08HB6* positively regulates the expression of the *BnaABF4* and *BnaDREB2A* genes under dehydration and salt stress. We conclude that four *BnaHB6* homologues have distinct functions in response to stress despite high sequence similarity, possibly indicating different binding preferences with *BnaABF4* and *BnaDREB2A*. We hypothesize that *BnaC08HB6* and *BnaA09HB6* function in a complex regulatory network under stress.

**Supplementary Information:**

The online version contains supplementary material available at 10.1007/s11103-024-01465-6.

## Key message

*BnaHB6* homologues show different response to abiotic stress and light conditions and differential transcriptional regulation of *BnaABF4* and *BnaDREB2A* target genes under dehydration and salt stress.

## Introduction

The regulation of gene transcription is a highly complex process in which transcription factors (TFs) act as regulatory proteins to activate or repress transcription by binding to specific DNA sequences in promoters of target genes. In *Arabidopsis thaliana*, about 40% of the identified TFs, including HD-Zip, NAC, AP2/EREBP, GARP, ARF, SBP, MADS-box, ABI3/VP1 and WRKY, are plant-specific genes.

Plant-specific homeodomain-leucine zipper (HD-Zip) TFs are important regulators of growth and development as well as response to stress that control various signaling pathways through interactions with other genes (Ariel et al. 2007). A highly conserved 60-amino acid homeodomain (HD) and the adjacent less conserved leucine zipper motif (LZ) play an important role in this process (Henriksson et al. 2005). The HD-Zip gene family is divided into four subfamilies: HD-Zip I, HD-Zip II, HD-Zip III and HD-Zip IV, which differ in their DNA binding specificity, gene structures and biological functions (Henriksson et al. 2005; Ariel et al. 2007). Among them, *HD-Zip I* genes are involved in developmental processes such as root and stem elongation, leaf morphology determination, flowering and pollen hydration, and stress response (Wang et al. 2003; Henriksson et al. 2005; Ariel et al. 2007; Ré et al. 2014; Ribone et al. 2015). In general, the members of the HD-Zip I subfamily show structural similarity and functional redundancy (Sessa et al. 1993; Palena et al. 1999; Johannesson et al. 2001; Żyła and Babula-Skowrońska 2023, for review). However, more detailed molecular studies have revealed their functional diversity in the regulation of biological processes, as evidenced by altered expression patterns in response to external stimuli, divergence in binding sites, differences in interaction with other proteins and the specificity of their site activity (in organs or tissues) (Henriksson et al. 2005; Ré et al. 2014; Capella et al. 2015; Perotti et al. 2019, 2021). One of the mechanisms responsible for this are changes within the LZ of certain HD-Zip I proteins, which can influence the efficiency and specificity of the DNA-binding process. It is also hypothesized that changes in regulatory motifs outside of HD and LZ involved in protein-protein interactions may be a source of functional diversity between evolutionarily related *HD-Zip I* genes (Olsson et al. 2004; Arce et al. 2011; Capella et al. 2014; Ré et al. 2014; Ribone et al. 2015). For example, the paralogous genes *AtHB7* and *AtHB12* in *A. thaliana* are upregulated under drought stress and in response to ABA, but they show specific, non-overlapping functions in root development; only *AtHB7* is involved in root growth at early stages of plant development (Olsson et al. 2004; Ré et al. 2014). In contrast to AtHB7 and AtHB12, AtHB6 has been described as a negative regulator of the ABA signaling pathway that physically interacts with ABI1 (serine/threonine protein phosphatase 2C) to act downstream under drought stress (Söderman et al. 1999; Himmelbach et al. 2002; Henriksson et al. 2005).

Many orthologs of *ATHB* genes from *A. thaliana* have been found in other plant species such as sunflower, tomato, cucumber, cotton, rice and *Brassica* (Lin et al. 2008; Manavella et al. 2008; Ariel et al. 2010; Liu et al. 2013; Zhang et al. 2016; Yang et al. 2020; Peng et al. 2022). Despite their evolutionary relationship, they show only partial functional similarity at the transcriptional level. Many of them have evolved novel tissue preferences that have led to changes in morphological traits to facilitate adaptation to different environmental conditions.

Oilseed rape (*Brassica napus* L.) is an allopolyploid species whose genomes have evolved by whole-genome triplication (Parkin et al. 2005). Many genes in these plants are preferentially maintained in a duplicated state with very similar sequences, but they have gradually changed their functions by sub- or neofunctionalization (Chalhoub et al. 2014; Babula-Skowrońska et al. 2015; Jiang et al. 2015). Recently, the expansion and expression patterns of *HD-Zip* genes in the *Brassica* species have been documented under different abiotic stress conditions (Jing et al. 2017; Khan et al. 2018; Peng et al. 2022; Yin et al. 2023). These studies showed dynamic changes in the expression patterns of homeobox genes in different tissues in relation to their response to abiotic stress and in the distribution of functional domains. So far, only a few *HD-Zip I* genes have been identified in oilseed rape (*Brassica napus* L.), so their functions are still poorly known (Peng et al. 2022). Previous studies have shown that AtHB6 is involved in stress response processes in *A*. *thaliana* (Himmelbach et al. 2002; Henriksson et al. 2005). However, its role, particularly in the context of duplication, has not yet been described in the *Brassica* genus.

In this study, we characterized four *BnaHB6* homologues in *B. napus* in terms of their phylogenetic relationship, structure, transcriptional activity and expression patterns. Based on the functional differences in induction under dehydration and salt stress, we identified factors contributing to their retention in the genome. We found that only two of the four *BnaHB6* genes, namely *BnaA09HB6* and *BnaC08HB6* are strongly induced under dehydration and salt stress. Our studies have also revealed a novel antagonistic function of the gene pairs *BnaA09HB6*/*BnaC08HB6* and *BnaA04HB6*/*BnaC04HB6* under light conditions. Furthermore, we found that the evolutionarily related genes *BnaHB6*, *BnaHB5* and *BnaHB16* have partially overlapping functions in plant development, response to ABA and stress response. ChIP-qPCR analyzes and transcriptional profiling of *BnaHB6*s in WT plants and the *bnac08hb6* mutant revealed the role of *BnaA09HB6* and *BnaC08HB6* in regulating the expression of four *BnaABF4* and two *BnaDREB2A* homologues in response to stress through direct binding to their promoter regions. We conclude that *BnaA09HB6* and *BnaC08HB6* are transcriptional activators of some *BnaABF4* and *BnaDREB2A* genes under dehydration and salt stress.

## Materials and methods

### Plant material, growth conditions and stress treatments

*B. napus* cv Monolit (Plant Breeding Strzelce Ltd., Co.) and *A*. *thaliana* Heyhn ecotype Columbia (Col-0) were used in this study. Line G2000-375-1 from the *B. napus* TILLING population with point mutations in the *BnaC08HB6* gene was kindly provided by Prof. Ian Bancroft (University of York, United Kingdom). All plants were grown on soil or Hoagland medium in a controlled growth chamber at 20/18 °C under long-day photoperiod (16-h/8-h light/dark cycle). Whole plants were harvested from the first to the fourth week of growth including cotyledons or true leaves, hypocotyl and roots for analysis and at fixed daily time points. Stress conditions were performed according to Babula-Skowrońska (2021) with minor modifications. For dehydration and salt stress, three-week-old oilseed rape plants were transferred to Hoagland medium containing 15% PEG8000 and 200 mM NaCl, respectively. For ABA treatment, three-week-old *B. napus* seedlings were transferred to Hoagland medium containing 50 µM ± ABA. For cold stress, three-week-old plants were placed at + 4 °C for 7 days. Leaves and roots were harvested 24 h or 7 days after treatment and then immediately frozen in liquid nitrogen. Circadian rhythm was analyzed in a 24-h cycle with 16 h of light and 8 h of darkness and samples were collected every two hours, from one hour after lights on (9:00 am) on day 0 to one hour before lights on (7:00 am) on day 1. All experiments were performed in three independent biological replicates.

### Isolation and cloning of the *BnaHB6*, *BnaHB5 *and *BnaHB16* genes

Total DNA and RNA were isolated from *B. napus* leaves using the DNeasy Plant Kit and the RNeasy Plant Mini Kit (Qiagen), respectively according to the manufacturer’s instructions. The genomic and full-length coding sequences of the *BnaHB6*, *BnaHB5* and *BnaHB16* genes were isolated using DNA and cDNA as templates (Table S1). The sequences obtained were cloned into the pENTR/D-TOPO vector and then subcloned into compatible vectors using the Gateway system. The primers used are listed in Supplementary Table S2. The *B. napus* genes are named according to the gene nomenclature system for the *Brassica* genus, which specifies the species-genome-gene name order (Ostergaard and King 2008).

### Phylogenetic tree reconstruction and bioinformatic analyses

The predicted amino acid sequences of HD-Zip I proteins of *A. thaliana* and *B. napus* were used to reconstruct the phylogenetic trees. First, these sequences were aligned using the Clustal Omega program and then the phylogenetic trees were reconstructed using the neighbor-joining algorithm implemented in MEGA11 (https://megasoftware.net/). A total of 1,000 bootstrap replicates were used to calculate the confidence level for each node. Motif and domain analyzes of BnaHB6 proteins were performed using the SMART program (http://smart.embl.de/smart/set_mode.cgi?NORMAL=1). Exon-intron analysis of *BnaHB6* genes was performed by aligning of the genomic and CDS sequences and then visualized using the GSDS gene structure display server (http://gsds.gao-lab.org/). Putative ABA-, phytohormone-, light- and stress-responsive *cis*-elements in approximately 1.5-kb promoter regions of *BnaHB6* genes were identified using the PlantCare database (https://bioinformatics.psb.ugent.be/webtools/plantcare/html/).

### Transcriptional activation assay in yeast cells

To study transcriptional activity, the coding regions of BnaHB6s were cloned separately into the pGBKT7 vector carrying the GAL4-responsive GAL1 promoter and the *HIS3* reporter gene and then independently introduced into yeast strain AH109 using the Yeastmaker™ Yeast Transformation System 2 (TaKaRa). The empty pGBKT7 vector was used as a negative control. The transformed yeasts with four BnaHB6 constructs were spotted on SD/-Trp and SD/-Trp/-Ade/-His/X-α-gal media and grown at 30 °C for 3 days to test the transcriptional activity. For the quantitative assay, β-galactosidase activity was monitored based on O-Nitrophenyl β-D-galactoside (ONPG) as a substrate in a liquid assay according to the manufacturer’s instructions (Clontech). The primers used are listed in Supplementary Table S2.

### Isolation and cloning of the *BnaHB6* promoter regions

Over 1.5-kb upstream of the start codon of the *BnaHB6* genes were isolated from *B. napus* genomic DNA and cloned separately into the pBract104-GUS vector (BRACT, John Innes Centre, UK) and verified by sequencing (pro*BnaHB6*::GUS). The recombinant vectors were individually introduced into *Agrobacterium tumefaciens* strain AGL-1 by electroporation and subsequently transformed into wild-type plants of *A. thaliana* (Col-0) by the floral dip method (Clough and Bent 1998). The putative transformants were selected by placing the seeds on agar-solidified ½ MS medium containing 50 mg l^−1^ kanamycin and the integrity of the transgenes was determined by PCR with specific oligonucleotides for each construct. Five to ten positive independent lines of T_3_ generation for each construct were used for the analyses. For dehydration and salt stress, two-week-old transgenic *A. thaliana* seedlings were placed on medium containing 15% PEG8000 and 200 mM NaCl, respectively for 12 h. For ABA treatment, two-week-old transgenic *A. thaliana* seedlings were placed on medium containing 50 µM ± ABA for 6 h. The untreated transgenic plants were used as controls. All primer sequences for vector construction are listed in Supplementary Table S2.

### Histochemical GUS staining

Histochemical GUS staining for the GUS activity assay in the transgenic *A. thaliana* plants was performed as described previously (Babula-Skowrońska et al. 2015). Whole seedlings or different tissues were immersed in the staining buffer containing 1 mM 5-bromo-4-chloro-3-inodyl-β-glucuronic acid (X-Gluc), 100 mM sodium phosphate (pH 7.0), 0.1 mM EDTA, 0.5 mM ferricyanide and 0.1% Triton X-100 at 37 °C for 6 h. The GUS-stained seedlings were washed in 70% ethanol. Three to five independent biological replicates were tested, each with three technical replicates.

### Isolation of total RNA, cDNA synthesis and quantification of gene expression by qRT-PCR

For gene expression, total RNA isolation and first-strand cDNA synthesis were performed as previously described (Babula-Skowrońska 2021). The qRT-PCR was carried out using the SYBR qPCR Mix (Promega) on the CFX96 real-time system (Bio-Rad) according to the manufacturer’s protocol. Reactions were performed according to the following program: 10 min at 95 °C, followed by 40 cycles at 95 °C for 15 s, 60 °C for 1 min and 72 °C for 1 min. Each experiment was performed with three biological replicates, each containing three technical replicates. Relative expression values were calculated using the 2^−ΔΔCt^ method with *PP2A* and *TIP41* selected as endogenous reference genes by Chen and coworkers (2010). Significance was determined using Student’s t-test. The gene-specific primer sequences used in this work are listed in Supplementary Table S2.

### ChIP-qPCR analysis

The CDS of the *BnaHB6* genes were cloned into the binary plant vector pEarlyGate103-His under the control of the CaMV35S promoter to obtain constructs for ChIP-qPCR analysis. The 35S::BnaHB6-His constructs were transiently transformed into *B. napus* leaves according to the protocol of Mooney and Graciet (2020). Genomic DNA was isolated and cross-linked with 1% formaldehyde, quenched with 134 mM glycine and frozen at - 80 °C. Chromatin was disrupted into 0.2-0.5 kb fragments by sonication. Immunoprecipitation followed by quantitative PCR (qPCR) was performed using commercially available anti-His (Cell Signaling Technology). The 10% of the supernatant before the addition of the antibody was used as DNA input control for the qPCR reaction. The enrichment of the *BnaABF4* and *BnaDREB2A* promoters by *BnaA09HB6* and *BnaC08HB6* was calculated by comparing the DNA immunoprecipitated with the anti-His antibody to the input DNA. The primers are listed in Supplementary Table S2.

## Results

### Phylogeny and characteristics of the *BnaHB6 *homologues

**Fig. 1:**
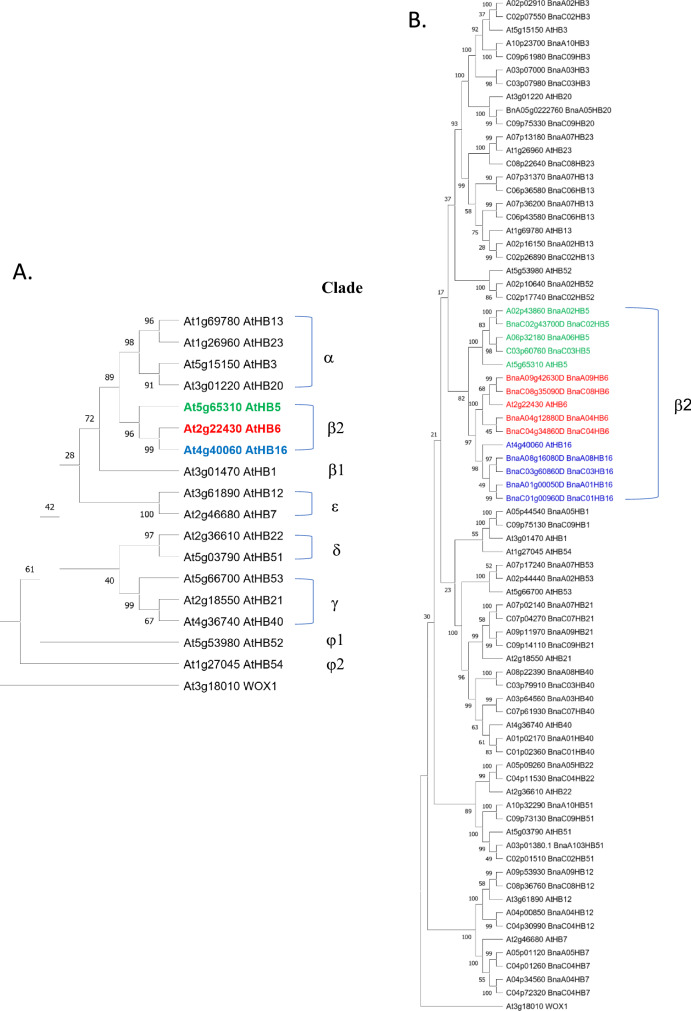
Phylogenetic analysis of the HD-Zip I proteins of *A. thaliana* and *B. napus*. Phylogenetic trees were constructed using the Neighbor-Joining method in MEGA11. Bootstrap values for 1000 replicates are given for each branch. **A.** The phylogenetic tree of the 17 HD-Zip I proteins of *A. thaliana*. The clades are labelled α, β1, β2, γ, δ, ε, φ1 and φ2 according to Henriksson et al. (2005). **B.** The phylogenetic tree of the HD-Zip I proteins of *A. thaliana* and *B. napus*

Based on syntenic gene analysis between *A. thaliana* and *B. napus* (http://brassicadb.cn/#/syntenic-gene/), we identified and cloned four *BnaHB6* homologues (*BnaA04HB6*, *BnaC04HB6*, *BnaA09HB6* and *BnaC08HB6*) from the *B. napus* genome. To investigate their evolutionary relationship, we constructed an unrooted phylogenetic tree encompassing all members of the HD-Zip I subfamily of *A. thaliana* and *B. napus* and confirmed the duplication events in the *Brassica* lineage that occurred during the evolution of Brassicaceae (Fig. 1A and 1B). In agreement with previous studies, the HD-Zip I proteins of *A. thaliana* are clustered into eight clades, with the AtHB6, AtHB5 and AtHB16 proteins located in a common clade β2 (Fig. 1A). Similar to *Arabidopsis*, the BnaHB6 proteins are also grouped with the BnaHB5 and BnaHB16 proteins in the same clade, confirming their close evolutionary relationship (Fig. 1B). BnaA09HB6 and BnaC08HB6 belong to the same cluster as AtHB6, while BnaA04HB6 and BnaC04HB6 are the more distant their paralogs, suggesting the *Brassica* lineage-specific whole genome duplication. These results were also confirmed by sequence analyses. The open reading frame (ORF) length of the *BnaHB6* genes ranges from 903 bp for *BnaA04HB6* and *BnaC04HB6* to 936 bp for *BnaA09HB6* and *BnaC08HB6*, and they encode polypeptides between 300 and 311 amino acids. The sequence similarity of these genes ranges from 77.8% between *BnaA04HB6* and *BnaA09HB6* at the genomic level to 96.8% between BnaA04HB6 and BnaC04HB6 at the amino acid level (Fig. S1A). All *BnaHB6* genes have a similar structure with three exons and two introns (Fig. S1B). Motif analysis of the BnaHB6 proteins confirmed the presence of the highly conserved HD and LZ as well as the putative phosphorylation sites (Ser, Thr and Tyr) (Fig. S1C). However, the N- and C-terminal regions of these proteins are more divergent in sequence and have a different arrangement of additional regulatory motifs previously reported in *Arabidopsis* AtHB6 (Arce et al. 2011; Fig. S1C and S1D). We found similar motif compositions in AtHB6, BnaA09HB6 and BnaC08HB6, suggesting their functional similarity (Fig. S1C). In contrast, only one and two motifs were conserved in BnaA04HB6 and BnaC04HB6, respectively. These differences are due to a single-nucleotide polymorphism and could influence their functional specificity.

### *BnaHB6 *act as transcriptional activators

**Fig. 2:**
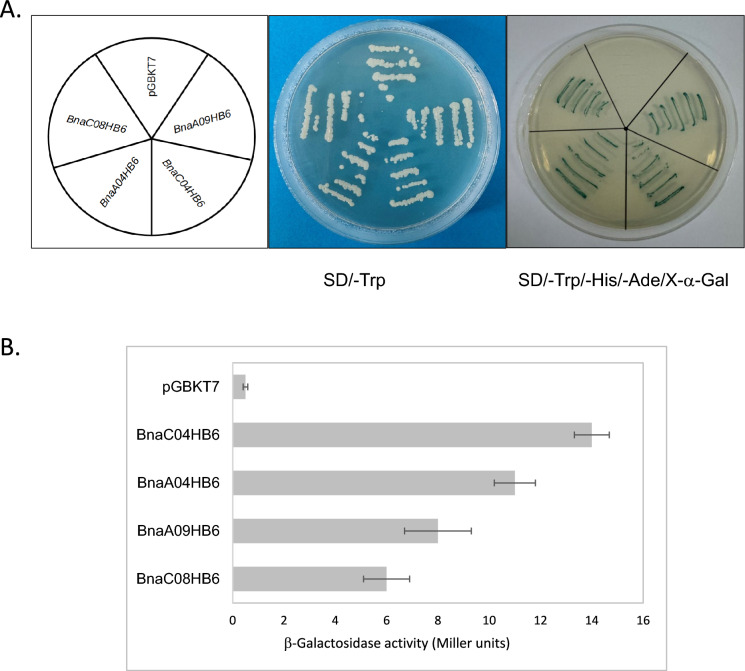
Transcriptional activity of the BnaHB6 proteins in yeast cells. The full length coding sequences of BnaHB6s were fused to the GAL4 DNA-BD and transformed into yeast strain AH109. **A**. Transformants were plated out on SD/-Trp (control medium) and SD/-Trp/-Ade-/-His/X-α-Gal (selection medium). Yeast cells expressing GAL4DBD were used as negative control; **B**. Quantitative β-galactosidase assay with ONPG as substrate. The β-Gal activity was determined as Miller units. The error bars represent three technical replicates

To investigate the transcriptional activity of BnaHB6s, their full-length coding sequences were fused to the GAL4 DNA-binding domain (BD) and then transformed into yeast strain AH109. The BnaHB6 transformants were tested for their ability to activate GAL4 transcription and promote yeast growth in the selective media. They grew well on SD/-Trp medium and showed α-galactosidase activity on SD/-Trp-His-Ade, except for the pGBKT7 empty vector (Fig. 2A). These results were further confirmed by β-galactosidase activity assay (Fig. 2B). This indicates that BnaHB6 are transcriptional activators in yeast and are therefore likely capable of regulating downstream gene expression as TFs.

### Expression profiling of *BnaHB6*, *BnaHB5 *and *BnaHB16 *genes

**Fig. 3:**
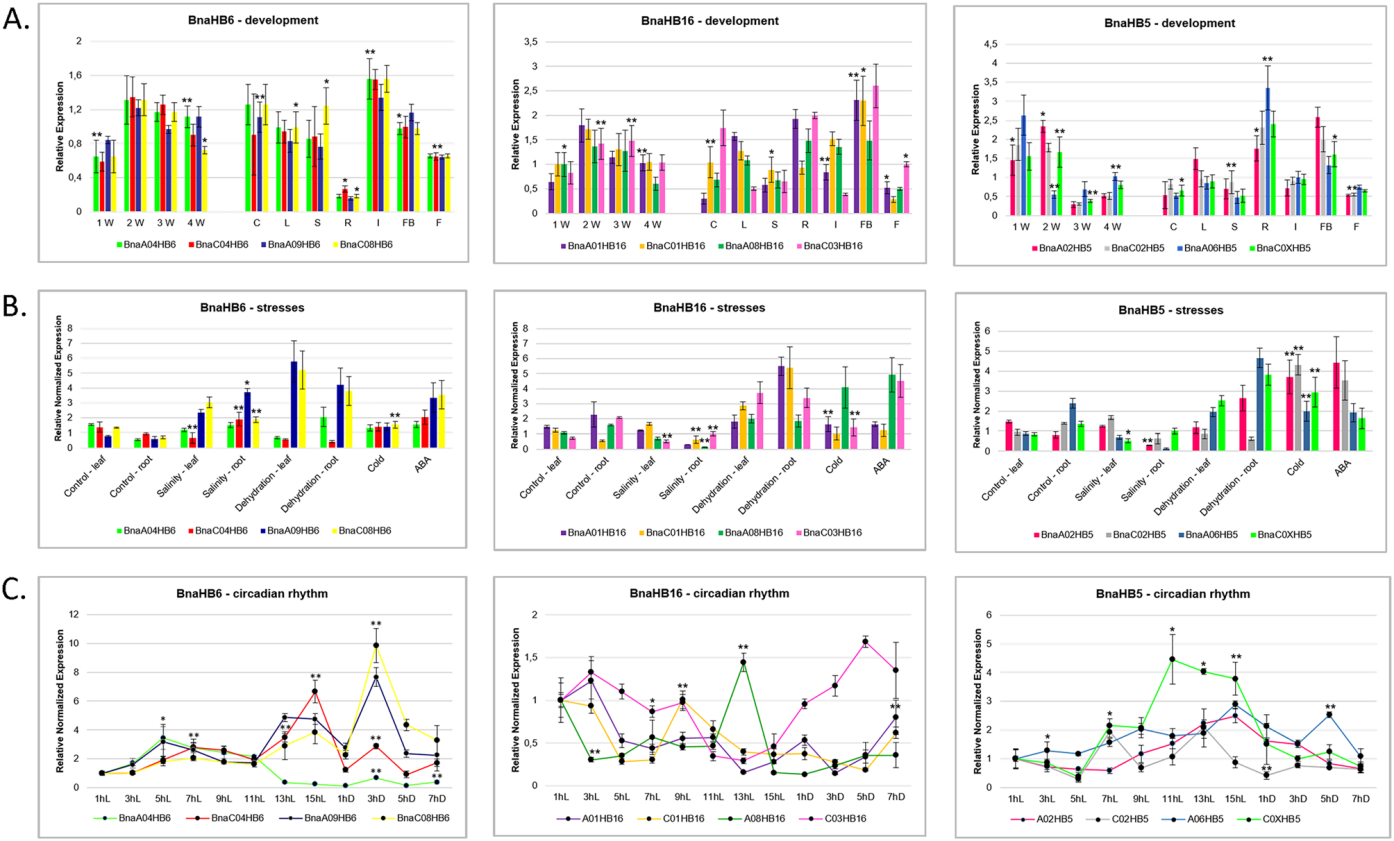
Expression profiles of the *BnaHB6* homologues and their closely related *BnaHB5* and *BnaHB16* genes at early developmental stages, in different tissues of *B. napus* plants and in response to abiotic stresses and light conditions. **A**. RT-qPCR analysis of *BnaHB6*, *BnaHB5* and *BnaHB16* transcripts at early developmental stages and in different tissues. Whole plants from the first to fourth week of growth with cotyledons or true leaves, hypocotyl and roots were harvested for analysis; C-cotyledons, L-leaf, S-stem, R-root, I-inflorescence, FB-flower buds and F-flowers. Values are relative to the expression level of the *PP2A* and *TIP41* reference genes; **B**. RT-qPCR analysis of *BnaHB6*, *BnaHB5* and *BnaHB16* transcripts in leaves and roots under different abiotic stresses and ABA treatment. The expression patterns of these genes were determined after NaCl (200 mM), dehydration (15% PEG8000), cold stress (+ 4 °C) and exogenous ABA treatment (50 µM ± ABA); **C.** RT-qPCR analysis of the transcript of *BnaHB6*, *BnaHB5* and *BnaHB16* in a 24-h cycle with 16 h of light and 8 h of darkness. Arabic numerals with L or D below each line indicate the duration of light or dark exposure in hours. The error bars show the standard deviation (SD) for three replicates (n = 3). Asterisks indicate statistically significant differences compared to the untreated control, calculated with the Student’s t-test (*P < 0.005; **P < 0.001)

In general, evolutionarily related genes may have similar functions. To understand the functional relationship between the four *BnaHB6* homologues, we analyzed their expression patterns in different tissues, circadian rhythm, at early developmental stages and under dehydration, salt and cold stresses, and exogenous ABA treatment (Fig. 3). The *BnaHB6* genes are expressed in all tissues examined, with high expression in inflorescences and much lower in roots than in other tissues (Fig. 3A). Moreover, the expression of *BnaHB6* genes increases rapidly during the first two weeks of plant growth and there are no significant differences in transcript levels between them (Fig. 3A). This indicates that they are functionally redundant in plant growth and development. In contrast, these four *BnaHB6* genes show different expression patterns under dehydration and salt stress (Fig. 3B). Two of them, namely *BnaA09HB6* and *BnaC08HB6*, are strongly induced in both leaves and roots under these stress conditions. In addition, the expression of *BnaA04HB6* increases in roots in response to dehydration. In contrast, the expression of all *BnaHB6* genes is not significantly altered under cold conditions, but *BnaA09HB6* and *BnaC08HB6* show slightly increased expression. All *BnaHB6* genes are induced under exogenous ABA treatment, but the genes regulated by stress conditions show higher expression (Fig. 3B). These results suggest that the orthologous genes *BnaA09HB6* and *BnaC08HB6* exhibit functional redundancy in the ABA-dependent signaling pathway under dehydration and salt stress. Interestingly, the gene pairs *BnaA04HB6*/*BnaC04HB6* and *BnaA09HB6*/*BnaC08HB6* show opposite fluctuations in expression under light conditions (Fig. 3C). We found that the expression of the *BnaA09HB6* and *BnaC08HB6* genes generally does not change during light exposure except between 11 and 15 h. However, they show increased expression with a maximum in the fifth hour of darkness.

As the phylogenetic analysis of HD-Zip I members confirmed the close relationship between the BnaHB5, BnaHB6 and BnaHB16 proteins, which may indicate their functional redundancy, we compared their expression profiles in different tissues, early developmental stages, stress conditions and circadian rhythm (Fig. 3). The *BnaHB5* and *BnaHB16* genes show partially different expression patterns compared to the *BnaHB6* genes under the investigated conditions. In particular, the *BnaHB5* and *BnaHB16* are highly expressed in roots and under cold stress, in contrast to the *BnaHB6* genes. However, we found that the expression profiles of *BnaA09HB6* and *BnaC08HB6* correlate with those of *BnaA01HB16*, *BnaC01HB16*, *BnaA06HB5* and *BnaC0XHB5* under dehydration conditions (Fig. 3B). The different expression patterns of the *BnaHB6*, *BnaHB5* and *BnaHB16* genes are observed in the circadian rhythm (Fig. 3C). Interestingly, the orthologous genes *BnaA08HB16* and *BnaC03HB16* show opposite induction in response to light conditions. *BnaA08HB16* is induced in light and its expression is similar to that of *BnaC04HB6* but is accelerated by two hours (Fig. 3C). In contrast, *BnaC03HB16* is induced in the dark and under dehydration, similar to the orthologous genes *BnaA09HB6* and *BnaC08HB6*, but its maximal expression is delayed by two hours.

### Analysis of *cis*-acting regulatory elements in the promoters of the *BnaHB6* genes and GUS activity of *BnaHB6* in the *A. thaliana* background

**Fig. 4:**
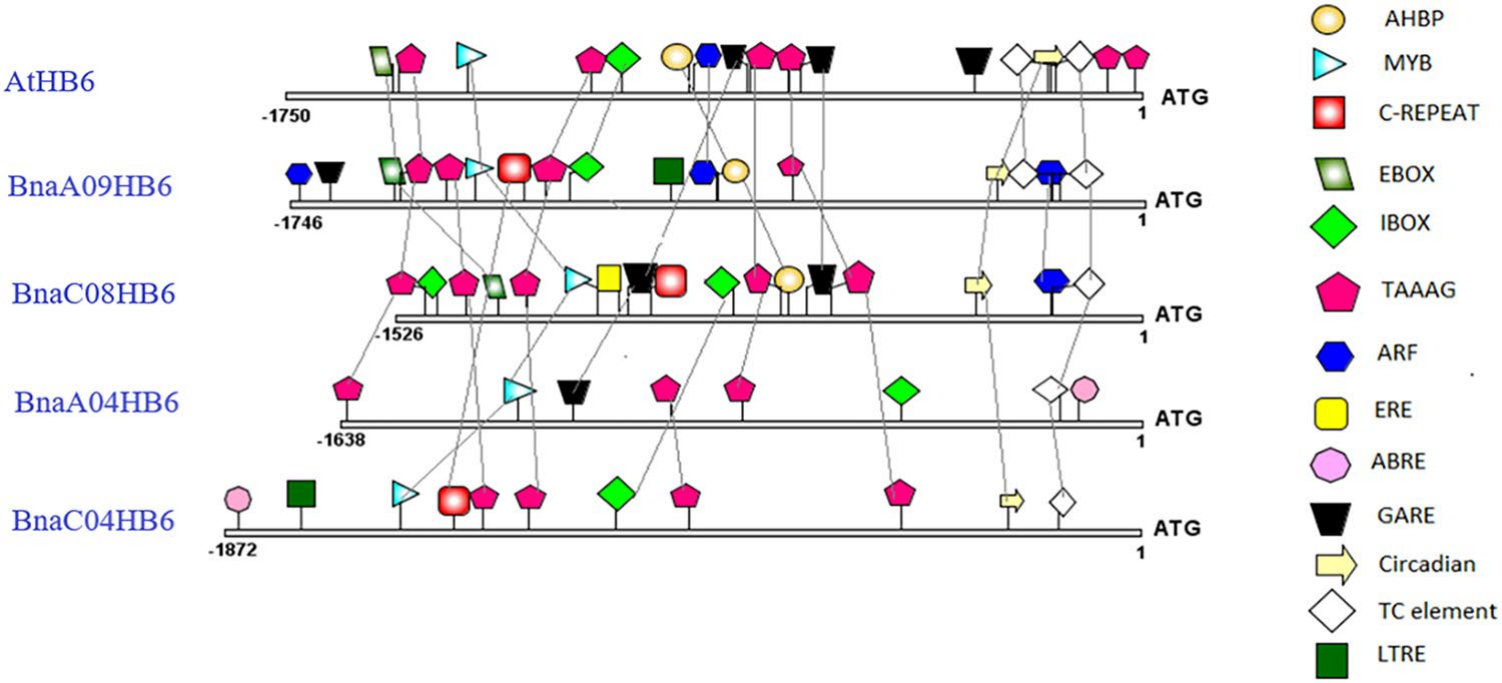
Comparison of the positions of putative transcription factor-binding sites in over 1.5 kb upstream of the transcription start site of *AtHB6* and four *B. napus BnaHB6* genes. The positions of the binding sites are indicated. The legend with symbols representing potential transcription binding motifs is on the right. The dashed lines connect the *cis*-acting elements located at conserved positions in the promoters of the *AtHB6* and four *BnaHB6* genes

**Fig. 5:**
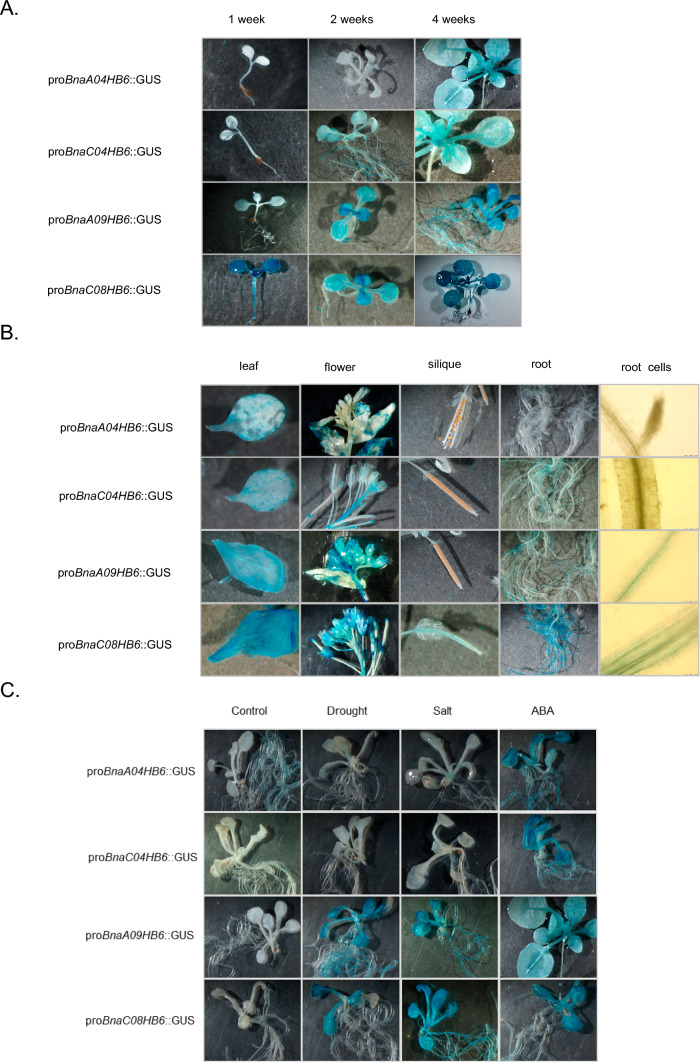
GUS staining and activity analysis of pro*BnaHB6*::GUS in transgenic *A. thaliana* plants: **A**. in early developmental stages; **B**. in various tissues such as leaves, flowers, siliques, roots and also in root cells; **C.** in two-week-old transgenic *A. thaliana* plants under dehydration, salt stress and ABA treatment. The expression of the *BnaHB6* genes was determined by histochemical GUS staining. Seedlings were placed in medium containing 15% PEG8000 and 200 mM NaCl for 12 h for dehydration and salt stress, respectively. For ABA treatment, two-week-old transgenic *A. thaliana* seedlings were placed in a medium containing 50 µM ± ABA for 6 h

To understand the mechanisms responsible for the differences in expression patterns of the *BnaHB6* homologues under stress and light conditions, we cloned 1.5-kb upstream promoter regions of all *BnaHB6* genes and then analyzed them for the location of *cis*-acting elements associated with response to stress, phytohormones and light-relevant factors using the PlantCARE database. These included the well-characterized elements: the ABA-responsive element (ABRE), MYB binding sites (MYB), auxin response factor, circadian-regulated element (Ebox), light-regulated element (Ibox), AHBP, ethylene-responsive element, circadian, low temperature-responsive element, defense and stress-responsive element (TC element), gibberellin-responsive element, C-repeat/dehydration-responsive element and TAAAG (Fig. 4). We found that stress-responsive *cis*-elements were more abundant in the promoters of the *BnaA09HB6* and *BnaC08HB6* genes than in the *BnaA04HB6* and *BnaC04HB6* genes, consistent with their expression patterns under stress conditions (Figs. 3 and 4). The arrangement of stress-response elements is conserved in the promoters of *AtHB6*, *BnaA09HB6* and *BnaC08HB6* confirming their close evolutionary relationship and similar expression patterns under stress conditions. To confirm the transcriptomic studies, we generated transgenic *A. thaliana* plants expressing the *β-glucuronidase* (*uidA*) reporter gene (GUS) under the control of the respective *BnaHB6* promoters (pro*BnaHB6*::GUS). We observed differences in the GUS activity of *BnaHB6*s at early developmental stages and in the tissues examined, with the activity of the *BnaC08HB6* promoter being generally higher (Fig. 5A and 5B). The *BnaA09HB6* and *BnaC08HB6* promoters are expressed at all early developmental stages, whereas the *BnaA04HB6* promoter shows no expression in one- and two-week-old seedlings. *BnaC08HB6* is preferentially expressed in siliques, but the GUS signal is not detected in flowers, siliques and roots in pro*BnaA04HB6*::GUS plants. The specific expression patterns for individual *BnaHB6* are observed in the root section. The *BnaA09HB6* and *BnaC08HB6* promoters show significant GUS activity in the columella root cap and root core including the xylem, while the *BnaC08HB6* promoter is additionally expressed in the endodermis and cortex. Subsequently, these transgenic plants were subjected to NaCl, PEG and ABA treatments to investigate their effects on the *BnaHB6* promoter activities (Fig. 5C). In pro*BnaA09HB6*::GUS and pro*BnaC08HB6*::GUS plants, induction of GUS was found in response to dehydration and salt stress. All *BnaHB6* plants showed GUS activity after treatment with ABA. These results confirm the expression patterns observed under stress conditions and only partially at different developmental stages and on different organs by qRT-PCR (Fig. 3).

Taken together, these results suggest that the *BnaHB6* TFs of *B. napus* are involved in different ways in the transcriptional control of developmental processes and stress responses.

### The role of the *BnaA09HB6* and *BnaC08HB6* orthologs in regulating the expression of the selected *BnaABF4* and *BnaDREB2A* homologues under abiotic stress conditions

**Fig. 6:**
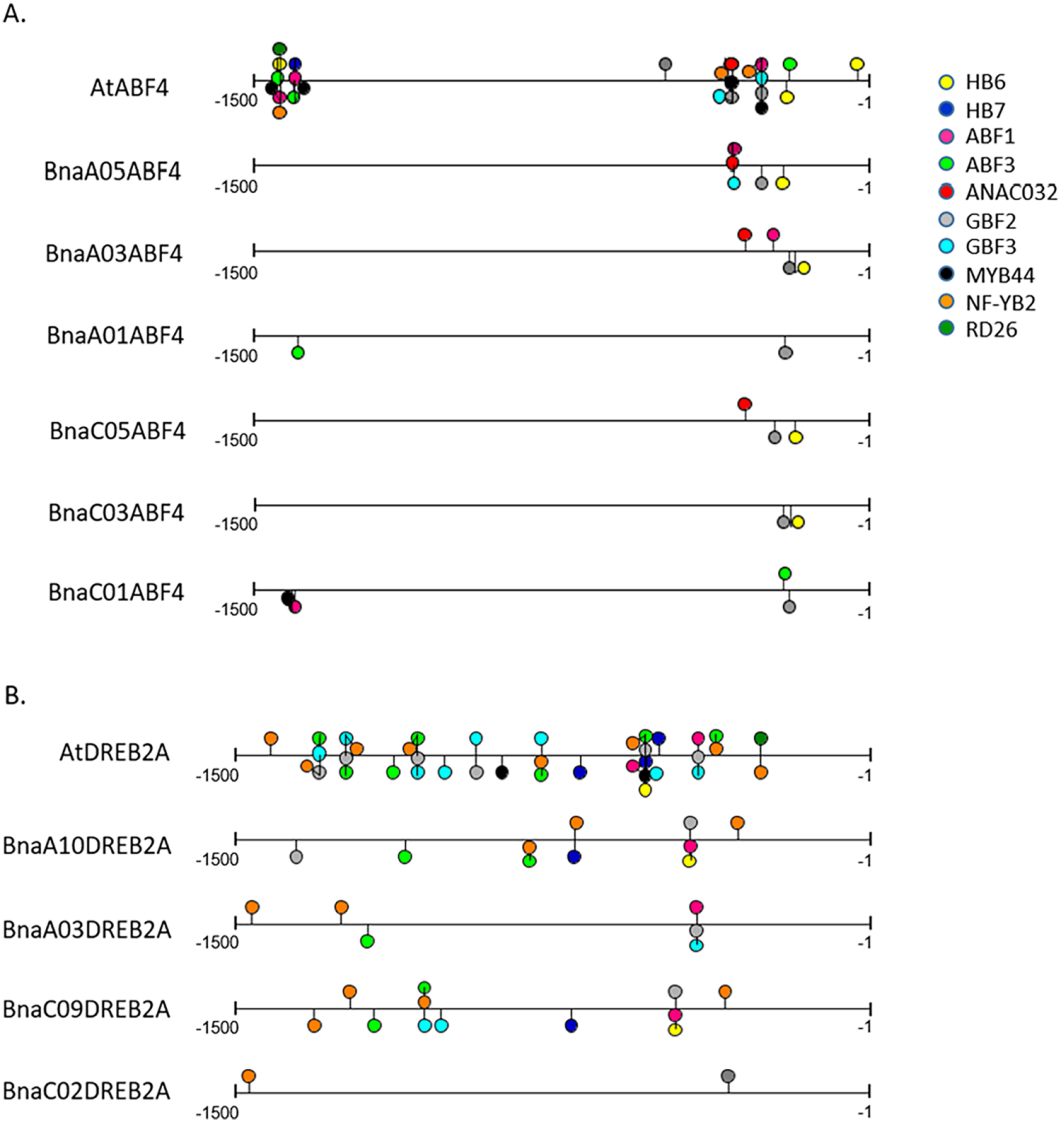
Schematic diagram of **A**. *AtABF4* and six *BnaABF4* promoters; **B**. *AtDREB2A* and four *BnaDREB2A* promoters, with TFs binding to motifs verified by ChIP-seq experiments and collected in the PlantPAN 3.0 database (Chow et al. 2019; http://plantpan.itps.ncku.edu.tw/). The positions of the predicted binding sites of TFs involved in resistance to osmotic stress by modulating ABA and salt responsiveness are indicated. The legend of the symbols representing potential transcription factors that bind to the sites is given on the right

**Fig. 7:**
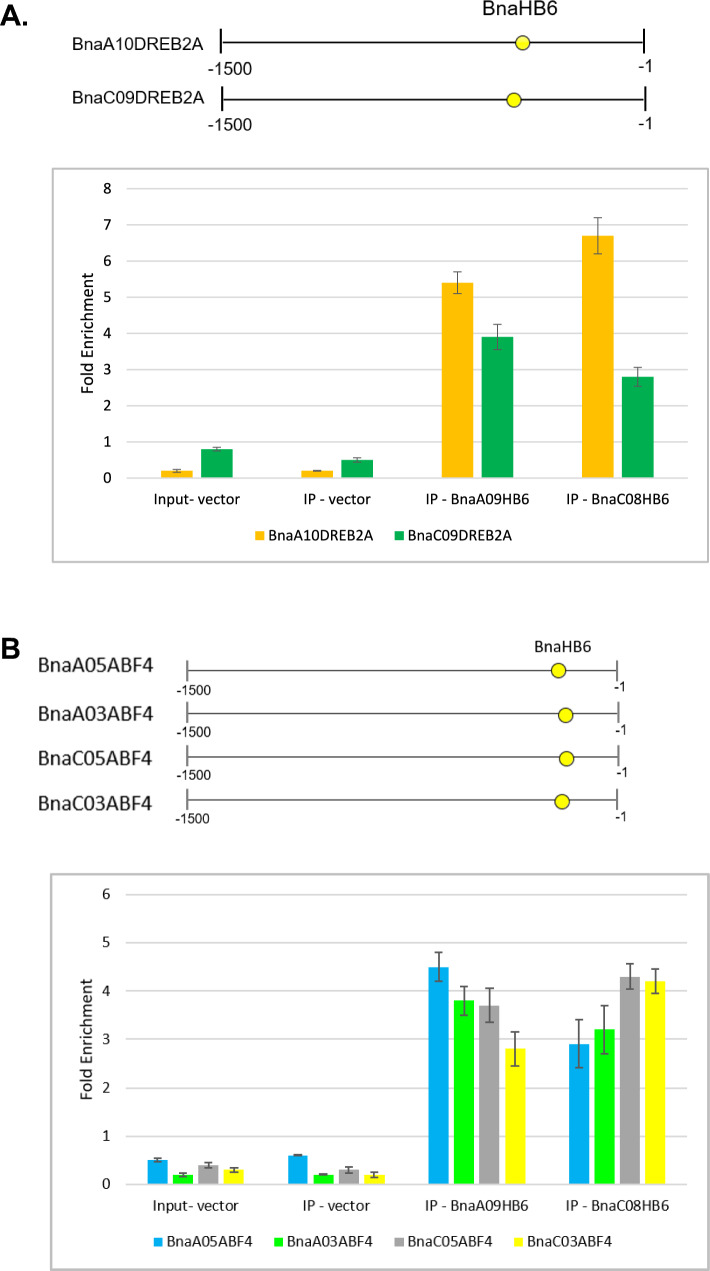
Characterization of *BnaABF4*-*BnaHB6* and *BnaDREB2A*-*BnaHB6* interactions by ChIP-qPCR analysis. **A**. Upper panel: schematic diagram of the promoters of *BnaA10DREB2A* and *BnaC09DREB2A* with the position of the binding motif recognized by *BnaA09HB6* and *BnaC08HB6*. Bottom: Enrichment of the promoter fragments of *BnaA10DREB2A* and *BnaC09DREB2A* with a motif recognized by two *BnaA09HB6* and *BnaC08HB6* in *B. napus*. **B.** Top: Schematic diagram of the promoters of *BnaA05ABF4*, *BnaA03ABF4*, *BnaC05ABF4* and *BnaC03ABF4* indicating the position of the binding element recognized by *BnaA09HB6* and *BnaC08HB6*. Bottom: Enrichment of promoter fragments of *BnaA05ABF4*, *BnaA03ABF4*, *BnaC05ABF4* and *BnaC03ABF4* with a motif recognized by two *BnaHB6* in *B. napus*. Sonicated chromatin was immunoprecipitated with anti-His antibodies. Fragmented genomic DNA was eluted from the protein-DNA complexes and subjected to qPCR analysis. The degree of enrichment of precipitated DNA fragments restricted to regions containing the motif under the study was quantified by qRT-PCR with specific primers. Fold enrichment for each promoter was normalized to the input controls. Vectors were performed as negative controls. The error bars show the standard deviation (SD) for three replicates (n = 3)

To gain insight into the role of *BnaHB6* genes in transcriptional regulation of genes under stress, we investigated their interactions with other stress-induced genes. Since the expression patterns of *AtHB6*, *BnaA09HB6* and *BnaC08HB6* are similar under stress, we hypothesized that their role in the expression regulation of target genes is evolutionarily conserved. First, we selected a group of *A. thaliana* genes involved in drought and salt stress response and controlled by *AtHB6* TF from the data collected in PlantPAN 3.0 (http://plantpan.itps.ncku.edu.tw/get_promoter_analysis.php). Among the experimentally verified genes involved in osmotic stress that have *AtHB6*-binding sites in their promoters, we selected *AtABF4* (ABRE Binding Factor 4) and *AtDREB2A* (Dehydration-Responsive Element Binding Protein 2) as possible downstream targets of *AtHB6* (Chow et al. 2019). In the *B. napus* genome, *BnaABF4* and *BnaDREB2A* are represented by multiple copies, i.e. six and four duplicates, respectively. We compared the promoter sequences of *AtABF4* and *AtDREB2A* with *BnaABF4* and *BnaDREB2A* homologues, respectively, to identify regions with conserved TF-binding sites (Fig. 6). We found the conserved position of the *HB6*-binding site in the promoters of four *BnaABF4* and two *BnaDREB2* homologues. To verify whether the *BnaA09HB6* and *BnaC08HB6* TFs directly bind to the fragment containing the CAATAATTG *cis*-element in these promoters of *BnaABF4* and *BnaDREB2A *in vivo, we performed ChIP-qPCR analysis. Our results showed significant enrichment of *BnaA09HB6* and *BnaC08HB6* in the promoters of four of the six *BnaABF4* genes and two of the four *BnaDREB2A* genes (Fig. 7A and 7B). We found that *BnaA09HB6* and *BnaC08HB6* enriched the two *BnaDREB2A* promoters by 3.9- to 5.4-fold and 2.8- to 6.7-fold, respectively. Similarly, *BnaA09HB6* and *BnaC08HB6* enriched the four *BnaABF4* promoters by 3.7- to 4.5-fold and 2.9- to 4.3-fold, respectively. In contrast, we found no significant enrichment of these two *BnaHB6* by other *BnaABF4* and *BnaDREB2A*. These results support the conclusion that the *BnaA10DREB2A*, *BnaC09DREB2A*, *BnaA09ABF4*, *BnaA03ABF4*, *BnaA05ABF4* and *BnaC05ABF4* genes are direct downstream targets of *BnaA09HB6* and *BnaC08HB6 *in vivo.

**Fig. 8:**
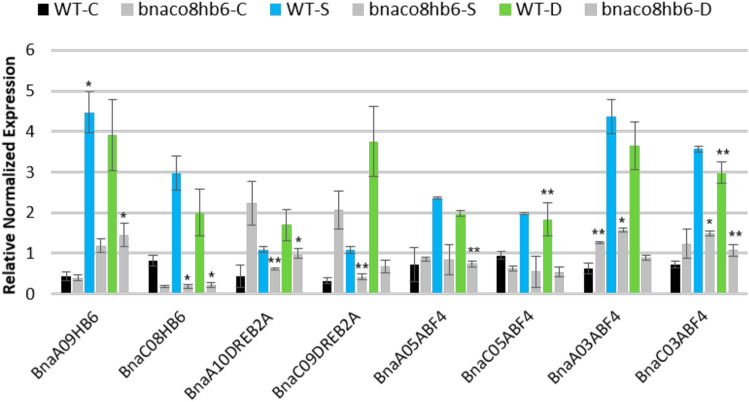
Expression patterns of *BnaA09HB6*, *BnaC08HB6*, *BnaA10DREB2A*, *BnaC09DREB2A*, *BnaA05ABF4*, *BnaC05ABF4*, *BnaA03ABF4* and *BnaC03ABF4* in WT and the *bnac08hb6* mutant under dehydration and salt stress. The error bars show the standard deviation (SD) for three replicates (n = 3). Asterisks indicate statistically significant differences calculated with the Student’s t-test (*P < 0.005; **P < 0.001)

The discovered interactions of some *BnaABF4* and *BnaDREB2A* homologues with *BnaA09HB6* and *BnaC08HB6* suggest that these genes may be involved in the same biological processes. To confirm their functional relationship, we examined their expression profiles under dehydration and salt stress in the wild type (WT) and the *bnac08hb6* TILLING mutant (Fig. 8). This line was selected on the basis of possible point mutations in the *BnaC08HB6* gene. Based on the SIFT program (http://sift.bii.a-star.edu.sg/; Ng and Henikoff 2003) (Ng and Henikoff 2003), we found five missense mutations (both non-conservative and conservative) and one nonsense mutation that could affect protein function (Fig. S2). After genotyping, the homozygous status of the *bnaco8hb6* mutant was confirmed with an abnormal phenotype (Fig. S3). We also observed that loss of BnaC08HB6 function increased the sensitivity of the plant to dehydration and the RWC (Relative Water Content) was lower in the *bnaco8hb6* mutant line compared to the WT (Fig. S4). The transcript level of *BnaC08HB6* was significantly reduced in the *bnac08hb6* mutant compared to the WT plants under both control and stress conditions (Fig. 8). The expression of *BnaA09HB6*, the orthologous gene of *BnaC08HB6*, is slightly reduced in the *bnac08hb6* mutant under unstressed conditions and significantly reduced under stress. In contrast, the expression of *BnaA10DREB2A*, *BnaC09DREB2A*, *BnaA05ABF4*, *BnaA03ABF4* and *BnaC03ABF4* increases in the *bnac08hb6* mutant compared to WT plants under control conditions. On the other hand, their expression is strongly reduced in the *bnac08hb6* mutant under salt stress and dehydration.

## Discussion

TFs are important regulators of gene expression that control complex biological processes. The *HD-Zip I* genes, which belong to the plant-specific *HD-Zip* gene family, are of great interest as they are involved in the regulation of plant growth and development and respond to stress (Henriksson et al. 2005). Among the 17 members of this subfamily in *A. thaliana* is the well-characterized *AtHB6* gene, which is a negative regulator of the ABA signaling pathway (Söderman et al. 1999; Himmelbach et al. 2002; Fig. 1A). In the present study, we identified and functionally characterized four *BnaHB6* homologues from *B. napus* that represent a lower number of duplicates than those resulting from genome triplication (Chalhoub et al. 2014). This is consistent with previous reports suggesting that some duplicates are lost in the long term due to fractionation followed by diploidization (Lynch and Conery 2000; Kumar et al. 2019; He et al. 2021). The phylogenetic analysis confirmed a close evolutionary relationship between four BnaHB6 homologous proteins and AtHB6, which are grouped in the same clade β2 (Fig. 1B). Among them, BnaA09HB6 and BnaC08HB6 belong to the same cluster as AtHB6, confirming their common origin and suggesting similar functions. BnaA04HB6 and BnaC04HB6, on the other hand, are distantly related to AtHB6 and probably originate from an additional whole genome triplication that occurred in the *Brassica*-specific lineage (The Brassica rapa Genome Sequencing Project Consortium et al. 2011). All BnaHB6 show transcriptional activity in yeast, so they may act as TFs to regulate downstream gene expression (Fig. 2). However, the differences in transcriptional activity between the orthologous gene pairs *BnaA04HB6/BnaC04HB6* and *BnaA09HB6/BnaC08HB6* were observed in the quantitative β-galactosidase assay. Previous studies have shown that changes in orthologous TFs contribute to the evolution of the regulatory network, affecting DNA-binding specificity and leading to phenotypic diversity (Price et al. 2007; Gabaldón and Koonin 2013; Nadimpalli et al. 2015). Therefore, further research should focus on investigating the regulatory interactions and expression patterns of these genes to understand their effects on phenotype. To determine the functional redundancy or diversity of the four *BnaHB6* homologues, we compared their sequences and motif organization with each other and with *AtHB6* and then studied their expression patterns in different tissues, at early developmental stages, under stress conditions and in circadian rhythm (Figs. 3 and S1). We observed differences in the sequences of introns and exons corresponding to the C- and N-terminal regions of the BnaHB6 proteins that affect the arrangement of regulatory motifs (Fig. S1). These changes may be responsible for their functional diversity, with the BnaHB6 homologues interacting with different target partners and regulating different signaling pathways. Previous reports have highlighted the likely role of additional regulatory motifs in the C- and N-terminal regions of HD-Zip I proteins in their functional divergence (Arce et al. 2011; Capella et al. 2014). Expression analyzes of the *BnaHB6* genes confirmed their functional complexity in growth and development processes as well as in the stress response (Fig. 3). They show similar expression patterns at early developmental stages and in the tissues studied, suggesting that they may have redundant functions in regulating these processes (Fig. 3A). On the other hand, only two of them, namely *BnaA09HB6* and *BnaC08HB6*, are involved in the response to dehydration and salt stress via the ABA signaling pathway and they are induced in the dark (Figs. 1B, 3B and 3C). Previous studies have found a link between the circadian clock and changes in the expression of genes involved in the response to abiotic stresses such as heat, cold and drought (Mizuno and Yamashino 2008; Wilkins et al. 2010). Our data confirm that the time of day of induction may be one of the factors responsible for the differential stress response of *BnaHB6* genes. The functions of some *HD-Zip I* genes in light-induced plant growth and development has been also postulated (Aoyama et al. 1995; Wang et al. 2003; Henriksson et al. 2005; Manavella et al. 2008; Choi et al. 2014). To date, the role of the *HB6* gene in the circadian rhythm and in the regulation of light-dependent developmental processes remains unknown.

Phylogenetic analyzes allowed us not only to determine the evolutionary relationship between the members of the gene subfamily, but also to speculate on their putative functions. Previous reports have shown that evolutionarily related *HD-Zip I* genes in different species (evolved from speciation) have similar functions in plant growth and development as well as in response to abiotic stress (Ariel et al. 2010; Harris et al. 2011; Wei et al. 2019; González et al. 2019). In *Arabidopsis*, *AtHB6* is located together with *AtHB16* and *AtHB5* in the clade β2 and they have evolved through gene duplications (Henriksson et al. 2005; Fig. 1A). In the *B. napus* genome, the *BnaHB6*, *BnaHB16* and *BnaHB5* genes each have four homologues are clustered in the clade β2, suggesting their functional redundancy in developmental processes and stress responses (Fig. 1B). Our data show the functional complexity of the *BnaHB6*, *BnaHB16* and *BnaHB5* genes at early developmental stages and under stress conditions with partially overlapping functions (Fig. 3). These results suggest that duplications play a role in the functional diversity of *BnaHB* genes belonging to the clade β2. This is consistent with previous studies showing functional divergence in members of other gene families such as PP2CA, CDPK and COL in the *Brassica* species (Ludwików et al. 2013; Zhang et al. 2014; Babula-Skowrońska 2021).

To better understand the mechanisms that regulate the expression of the *BnaHB6* homologues at the transcriptional level, we compared the number and localization of *cis*-regulatory elements in the *AtHB6* and the four *BnaHB6* promoters that might affect the expression activity and functional specificity of these genes in the stress response (Fig. 4). The observed differences in the number and localization of stress-responsible *cis*-acting elements in their promoters, with the promoters of *AtHB6*, *BnaA09HB6* and *BnaC08HB6* being more conserved, confirm that both *BnaHB6* genes are involved in the response to dehydration and salt stress. Using transgenic *A. thaliana* plants expressing the GUS reporter gene under the control of the individual *BnaHB6* promoter, we observed GUS activity of the orthologous genes *BnaA09HB6* and *BnaC08HB6* in the columella root cap and in the root core including the xylem (Fig. 5B). Previous molecular studies have shown that columella cells contain amyloplasts and can increase the root angle in the direction of gravity in root tips (Ge and Chen 2019). They are also involved in deepening the root structure during drought. The root xylem, on the other hand, is involved in the transport of water from the soil and from cell to cell, among other things.

In this study, we also gained insight into the role of *BnaHB6* genes of *B. napus* in the regulation of downstream target genes under dehydration and salt stress. Based on experimentally verified data on TF-binding sites in genes involved in drought, salt stress and ABA in *A. thaliana*, we selected *AtABF4* and *AtDREB2A*, whose promoters have *AtHB6*-binding sites (Chow et al. 2019). Consistent with the duplicated structure of the *B. napus* genome, the *BnaABF4* and *BnaDREB2A* genes are represented by a larger number of homologues, only some of which have *BnaHB6*-binding sites in their promoters (Fig. 6A and 6B).

We found that *BnaA09HB6* and *BnaC08HB6* bind directly to only four of six *BnaABF4* homologues and two of four *BnaDREB2A* to regulate their transcription (Fig. 7A and 7B). These results indicate that WGD, followed by genomic rearrangements, has led to changes in DNA-binding preferences. The discovered interactions between some homologues of *BnaABF4* and *BnaDREB2A* as well as *BnaA09HB6* and *BnaC08HB6* suggest that these genes may be involved in regulating the same biological processes. To determine the functional relationship between these genes, we examined their expression under dehydration and salt stress in the WT and the *bnac08hb6* TILLING mutant (Fig. 8). The *bnaco8hb6* mutant showed an abnormal phenotype with shortened root length and fleshy, thickened leaves (Fig. S3). In addition, loss of *BnaC08HB6* function increased the plant’s sensitivity to dehydration and decreased RWC compared to the WT (Fig. S4). Hence, *BnaC08HB6* is positively involved in the developmental processes independent of environmental changes and response to abiotic stress. We found that *BnaABF4* and *BnaDREB2A* as well as *BnaA09HB6* and *BnaC08HB6* were induced in the WT plants under both control and stress conditions and in the *bnac08hb6* mutant only under control conditions, with the exception of *BnaC05ABF4*. These results confirm that *BnaC08A09HB6* can positively regulate the expression of some *BnaABF4* and *BnaDREB2A* targets under stress conditions.

**Fig. 9:**
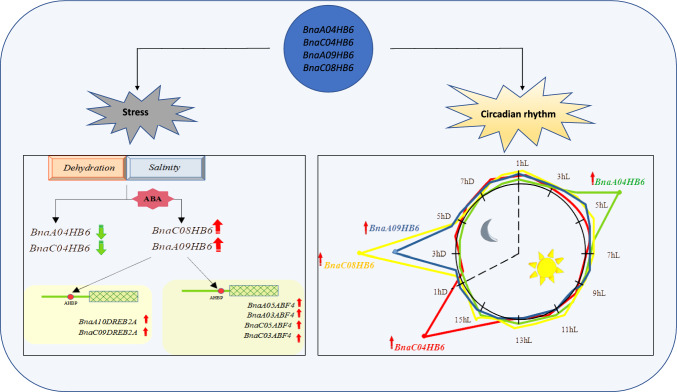
Working model of the functional diversity of the *BnaHB6* homologues in stress response and circadian rhythm and in the regulation of *BnaABF* and *BnaDREB2A* genes under dehydration and salt stress; Left: two of four *BnaHB6* homologues (*BnaA09HB6* and *BnaC09HB6*) are induced in an ABA manner under dehydration and salt stress and they directly upregulate the expression of some *BnaABF* and *BnaDREB2A* homologues under these stress conditions by binding to their promoters; Right: changes in the expression of four *BnaHB6* genes in a 24-h cycle with 16 h of light and 8 h of darkness

In conclusion, our results in this study provide new insights into the functional diversity of four *BnaHB6* homologues in regulating the stress response. First, we show the different transcriptional responses of the *BnaHB6* homologues under dehydration and salt stress as well as under light conditions (Fig. 9). We have identified *BnaC08HB6* and *BnaA09HB6* as molecular components acting downstream of the selected *BnaABF2* and *BnaDREB2A* in regulating the response to dehydration and salt stress. This is a first step towards understanding the differences in the gene regulatory network activated by individual *BnaHB6* homologues in response to stress.

### Supplementary Information

Below is the link to the electronic supplementary material.

Supplementary file1 (DOCX 15 KB)

Supplementary file2 (XLSX 16 KB)

Supplementary file3 (DOCX 483 KB)

Supplementary file4 (DOCX 72 KB)

Supplementary file5 (PPTX 4243 KB)

Supplementary file6 (PPTX 1846 KB)

## Data Availability

All data generated or analyzed during this study re included in this published article or as supplementary materials.
