# Supplementary material for: Functional and regulatory diversity of homeobox-leucine zipper transcription factors BnaHB6 under dehydration and salt stress in Brassica napus L
Source: Plant Mol Biol. 2024 May 15;114(3):59. doi: 10.1007/s11103-024-01465-6 (PMC11096223; doi:10.1007/s11103-024-01465-6)
Supplement: Supplementary file 1 — Supplementary file1 (DOCX 15 KB) [file 11103_2024_1465_MOESM1_ESM.docx]

The Supplementary Table S1: List of CDS sequences of the *BnaHB6*, *BnaHB5* and *BnaHB16* genes isolated in this study

>BnaA04HB6

ATGATGAAGAGATTAAGAAGTTCAGATTCAGTGGGTGGTCTGATCTCTTTATGTCACACATCTTCTACAGATGAGCAGAGTCCAAGAAGATACGGGTCGATGCTTGAAGGTTACGATGAGGATGAAGAAGAGGCGATAGCTGAGGAGAGAGGACAAACTGAGAAGAAGAGACGGTTAAGCATTAACCAAGTCAAAGCCCTGGAGAAGAACTTCGAGTTAGAGAACAAGCTTGAGCCTGAGAGGAAAGTGAAGTTAGCTCAAGAACTTGGTCTTCAAACTCGTCAAGTAGCTGTTTGGTTCCAGAACCGCCGTGCACGGTGGAAGACAAAACAGCTTGAGAAAGATTACGGTGTTCTTAAAACCCAGTACGATTCTCTCCGCCATAACTTTGATTCCCTCCGCAGTGACAATGAATCTCTTCTTCAAGAGATTAGTAAACTGAAGTCTAAGCTTAACGGAGGAGAAGAAGAAGAGGAAGAGAACAACGCCGTGGCGATGGAGAGTGATTTTTCCGTCAAGGAGGAAGAAGTTTCGCTGCCGGAAAATATCACAGAAGAACCGCCGTCGTCTCCTCCAGAGCTTCTAGAACATTCGGATAGTTTCAATTACCGGAGTTTTACCGATCTCCGGGATCTTCTTCCATTAAAGGCTGCGGCTTCTTCCTTCGCCGCTGCTGGATCGTCGGACAGCGGCGACTCGAGCGCCGTGTTGAACGAGGAGAGTAGCTCCAACGTCACGGTAACTCCGACGGCGGTTCCCGGCGGTAGTTTCTTCCAGTTTGTGAAAATGGAGCAGACGGAGGATCACGACGACTTTCTGAGTGGAGAAGAAGCTTGCGGTTTTTTCTCCGATGAACAGCCGCCGTCTCTACACTGGTACTCCGCCGTTGATCACTGGACTTGA

>BnaC04HB6

ATGATGAAGAGATTAAGAAGTTCAGATTCAGTGGGTGGTCTGATCTCTTTATGTCACACATCTTCTACAGATGAGCAGAGTCCAAGAAGATACGGGTCGATGCTTGAAGGTTACGATGAGGATGAAGAAGAAGCGATAGCTGAGGAGAGAGGACAAACTGAGAAGAAGAGACGGTTAAGCATTAACCAAGTCAAAGCCCTGGAGAAGAACTTCGAGTTAGAGAACAAGCTTGAGCCTGAGAGGAAAGTGAAGTTAGCTCAAGAACTTGGTCTTCAAACTCGTCAAGTAGCTGTTTGGTTCCAGAACCGCCGTGCACGGTGGAAGACAAAACAGCTTGAGAAAGATTACGGTGTTCTTAAAACCCAGTACGATTCTCTCCGCCATAACTTTGATTCCCTCCGCAGTGACAATGAATCTCTTCTTCAAGAGATTAGTAAACTGAAGTCTAAGCTTAACGGAGGAGAAGAAGAAGAGGAAGAGAACAACGCCGTGGCGATGGAGAGTGATTTTTCCGTCAAGGAGGAAGAAGTTTCGCTGCCGGAAAATATCACAGAAGAACCGCCGTCGTCTCCTCCAGAGCTTCTAGAACATTCGGATAGTTTCAATTACCGGAGTTTTACCGATCTCCGGGATCTTCTTCCATTAAAGGCTGCGGCTTCTTCCTTCGCCGCTGCTGGATCGTCGGACAGCGGCGACTCGAGCGCCGTGTTGAACGAGGAGAGTAGCTCCAACGTCACGGTAACTCCGACGGCGGTTCCCGGCGGTAGTTTCTTCCAGTTTGTGAAAATGGAGCAGACGGAGGATCACGACGACTTTCTGAGTGGAGAAGAAGCTTGCGGTTTTTTCTCCGATGAACAGCCGCCGTCTCTACACTGGTACTCCGCCGTTGATCACTGGACTTGA

>BnaA09HB6

ATGATGAAGAGATTAAGCAGTTCAGATTCAGTGGGTGGTCTCATCTCTTTATGTCCCACTACTTCCACAGATCAGCCGAGTCCAAGAAGATACGGGAGAGAGTTTCAGTCGATGCTTGAAGGTTACGAGGAGGAAGAAGAAGAAGCCGTAACCGAGGAAAGAGGACAAACCGGTTTAGCCGAGAAGAAGAGACGGTTAAGCATTAACCAAGTTAAAGCCTTGGAGAAAAATTTCGAGTTAGAGAACAAGCTTGAGCCCGAGAGGAAAGTGAAGCTAGCTCAAGAACTTGGTCTCCAACCTCGTCAAGTAGCTGTTTGGTTTCAGAACCGCCGTGCGCGGTGGAAGACAAAACAGCTCGAGAAAGATTACGGTGTTCTCAAAACGCAGTACGATTCTCTCCGCCATAACTTCGATTCCCTCCGCCGTGACAATGAATCTCTCCTTCAAGAGATCGGTAAACTAAAAGCTAAGCTAAACGGAGAAGAAGAAGTTGAAGAAGATGATGAAGATGAAGAGAACAACGCGGTGACGATGGAGTGTGATGTTTCCGTCAAGGAAGAAGAAGTTTCGTTGCCGGAGGAGCTTACAGATCCGCCGTCTTCTCCTCCGCAGCTTCTAGAACATTCCGACAGTTTCAATTACCGGAGTTTCACCGACCTCCGCGACCTTCTTCCGTTAAAGGCCGCGGCTTCCTCCGTCGCCGCCGCTGGATCGTCGGACAGTAGCGATTCGAGCGCCGTGTTGAACGAGGAAAGTAGCTCCAACGTTACGGCGGGTCCGGTGACTGTTCCCAGCGGTGGTTTCTTGCAGTTTGTGAAAATGGAGCAGACGGAGGATCACGACGACTTTCTGAGCGGAGAAGAAGCGTGCGGGTTTTTCTCCGATGAGCAGCCACCGTCTCTGCACTGGTATTCCACCGTTGATCAGTGGAACTGA

>BnaC08HB6

ATGATGAAGAGATTAAGCAGTTCAGATTCAGTGGGTGGTCTCATCTCTTTATGTCCCACTACTTCCACAGATCAGCCGAATCCAAGAAGATACGGGAGAGAATTTCAGTCGATGCTTGAAGGTTACGAGGAGGAAGAAGAAGAAGCCATAACCGAGGAAAGAGGACAAACCGGTTTAGCCGAGAAGAAGAGACGGTTAAACATTAACCAAGTTAAAGCCTTGGAGAAAAATTTCGAGTTAGAGAACAAGCTTGAGCCTGAGAGGAAAGTGAAGTTAGCTCAAGAACTTGGTCTCCAACCTCGTCAAGTAGCTGTTTGGTTTCAGAATCGCCGTGCGCGGTGGAAGACAAAACAGCTTGAGAAAGATTACGGTGTTCTCAAAACGCAATACGATTCTCTCCGCCATAACTTTGATTCCCTCCGACGTGACAATGAATCTCTTCTTCAAGAGATCGGTAAACTAAAAGCTAAGCTTAACGGAGAAGAAGAAGAAGAAGAAGATGTTGATGAAGAAGAGAACAACTTGGCGACGATGGAGAGTGATGTTTCCGTCAAGGAAGAAGAAGTTTCGTTACCGGAGCAGATCACAGAACCGCCGTCTTCTCCTCCGCAGCTTCTAGAGCATTCCGACAGTTTCAATTACCGGAGTTTCACCGACCTCCGCGACCTTCTTCCGTTAAAGACCGCGGCTTCCTCCGTCGCCGCCGCTGGATCGTCGGACAGTAGTGATTCGAGTGCCGTGTTGAACGAGGAAAGTAGCTCTAACGCTACGGCGGCTCCGGCGACGGTTCCCGGCGGCAGTTTCTTGCAGTTTGTGAAAATGGAGCAGACGGAGGATCACGACGACTTTCTGAGTGGAGAAGAAGCGTGCGGGTTTTTCTCCGATGAACAGCCACCGTCTCTGCACTGGTATTCCACCGTTGATCAGTGGAACTGA

>BnaA02HB5 ATGAAGAGATCACGAGGAAGCTCGGATTCTTTATCCGGTTTCTTACCAATTTATCACTCTACAGCAGACAAACAACTAAGTCCACGACCAACAGCCACCGGCTTTCTCTACCCCGGCAGCGCCGGAGACTACTCCCAGATGTTCGACGGTCTAGAAGAAGACGGAAGTCTAGAGGACATCGGCGTTGGACACGCGTCGTCTACGGCAGCAGCGGAGAAAAAACGGCGGTTGAGTGTAGTGCAAGTGAAAGCGTTAGAAAAGAATTTCGAGATTGATAACAAGTTAGAGCCCGAGAGGAAAGTGAAGCTGGCTCAAGAGCTTGGGCTGCAACCTCGACAAGTGGCGATCTGGTTTCAGAACCGCCGTGCTAGGTGGAAGACAAAGCAGCTCGAACGTGATTACGGCGTTCTTAAGTCAAACTTTGATTCACTCAAACGCAGCCGCGACTCGCTTCAACGTGATAACGATTCTCTTCTTGCAGAGATTAAAGAGCTGAGAGCAAAACTTGACGTGGAAGGGACATGCGGAAACAATGGTAACGCGGTGACAGAAGAAACCGGCGTTGTAAAACCGGTGGAAACGGTGGCGTTTCAGACGGTGATTGCTAATAACGAAGTCTTAGAGCTAAGCCAGTGTCCTCCACTGCCTGGGGAAGCTCCGGCATCGGAGCTCGCATACGAGATGTTTAGCATTTTCCCACGTACCGAAAGCTTCAGAGAAGATCCAGCCGACAGTAGCGACTCAAGCGCTGTTTTGAACGAAGAATATAGTCCTACGGCGGCGGCGGCGACAGCGGTCGAGATGTCGACGATGGGATGTTTTGGGCAGTTTGTGAAAATGGAAGAGCATGAAGATCTTTTTAGTGGAGAGGAAGCTTGCAAGTTGTTTGCGGATAATGAGCAGTGGTTTTGCTCTGGTCAGTGGAGTTCCTAA

>BnaC02HB5

ATGAAGAGATCACAAGGAAGCTCGGATTCTTTATCCGGTTTCTTACCAATTTGCCACTCTACAGCAGACAAACAACTAAGTCCACGTGCACGACCAACAGCCATCGGCTTTCTCTACCCCGGCGGCGCCGGAGACTACTCCCAGATGTTCGACGGTCTAGAAGAAGACGGAAGTCTAGAGGACATCGGCGTTGGACACGCGTCGTCTACGGCAGCAGCGGAGAAAAAACGGCGGTTGAGTGTAGTGCAAGTGAAAGCGTTAGAAAAGAATTTCGAGATTGATAACAAGTTAGAGCCTGAGAGAAAAGTGAAGCTGGCTCAAGAGCTTGGGCTGCAACCTCGACAAGTGGCGATCTGGTTTCAGAACCGCCGTGCTCGGTGGAAGACAAAGCAGCTCGAACGTGATTACGGCGTTCTCAAGTCAAACTTTGATTCACTCAAACGCAGCCGCGACTCGCTTCAACGTGATAACGATTCCCTTCTTGCAGAGATTAAAGAGCTGAGAGCAAAACTTGACGTGGAAGGGACATGCGGAAACAATGGTAACGCCGTGACAGAAGAAACGGACGTTGTAAAACCGGTGGAAACGGTCGCGTTTCAGACGGTGATTGCTAATAACGAAGTCTTAGAGCTAAGCCAGTGTCCTCCACTGCCTCGGGAAGCCCAGGCATCGGAGCTCGCATACGAGATGTTTAGCATTTTCCCACGTGCCGAAAGCTTCAGAGAAGATCCAGCTGATAGTAGCGACTCAAGCGCTGTTTTGAACGAAGAATATAGTCCCACGGCGGTGGCAGCGACAGCGGTTGAGATGTCGACGATGGGATGTTTTGGCCAGTTTGTGAAAATGGAAGAGCATGAAGATCTTTTTAGTGGAGAGGAAGCTTGCAAGTTGTTTGCGGATAATGAGCAGTGGTTTTGCTCTGGACAGTGGAGTTCCTAA

>BnaA06HB5

ATGAAGAGATCACGTGGGAGCTCCGATTCTTTATCCCGGTTCTTACCAATTTGCCACTCTGCAACCGACAATCAATTAAGTCCAAGACCAACAGCCACCGGCTTTCTCTATTCCGGTACCGGGGACTACTCCCCGATGTTTGACTGTCTAGAAGATGGAAGTCTAGAGGACATCGCCGTCGGACACGCGTCGTCTACGGCGGCAACGGAGAAAAAGCGGCGATTGCGTGTAGAACAAGTGAAAGCATTAGAGAAGAATTTCGAGATTGATAACAAGTTAGAGCCTGAGAGGAAAGTGAAACTGGCTCAAGAGCTCGGGCTGCAACCACGACAAGTGGCGATCTGGTTTCAGAACCGCCGTGCTCGGTGGAAGACAAAGCAGCTAGAGCGTGATTACGGTGTTCTCAAGTCAAACTTTGACTCACTCAAACGCAGCCGCGACTCCCTTCAACGTGATAACGATTCTCTCCATGCAGAGATTAAACAGCTGAGAGCAAAACTTAACGTGGACGGTATCAGCAGAAGCAGTAGTAACGCGTCGACGGAAGAAAACGTCTTAGTAAAGGCGGATGAAACGGTGATGCCTAGTAACAAAGTCTTAGAGCTAAACCAGCGTCCTTTGCCACCGCCACCACATATTCCTACGGCTACGGAAGCTCCGGCATTGGAGCTTGAATACGAGATGTTGAGCATTTTCCCACGTGCGGAGATCTTCAGAGAAGATCCTGCTGATAGTAGCGACTCAAGCGCTATTCTGAACGAGGAGTATAGTCCCACGGCGGCTGAAGCGGCCGCGGCTACGGCAGTTGAAATGTCGACGATGGGATGTTTTGGCCCATTTGTAAAAATGGAAGAGCATGAAGATCTTTTTAGTGGAGAGGAAGCTTGCAAGTTGTTTGCAGATAATGAGCAGTGGTATTGTTGA

>BnaC0XHB5

ATGAAGAGATCACGTGGGAGCTCCGATTCTTTATCCGGGTTCTTACCAATTTGCCACCCTGCAACAGACAATCAACTAAGTCCAAGACCAACAGCCACCGGCTTTCTCTATTCCGGTAGCGGAGACTACTCCCCGATGTTTGACTGTCTAGAAGATGGAAGTCTAGAGGACATCGCCGTCGGACACGCGTCGTCTACGGCGGCAACGGAGAAAAAGCGGCGATTGAGTGTAGAACAAGTGAAAGCATTAGAGAAGAATTTCGAGATTGATAACAAGTTAGAGCCTGAGAGGAAAGTGAAACTGGCTCAAGAGCTTGGGCTGCAACCACGACAAGTGGCGATCTGGTTTCAGAACCGCCGTGCTCGGTGGAAGACAAAGCAGCTTGAACGCGATTACGGTGTTCTCAAGTCAAGCTTTGACTCACTCAAACGCAGCCGCGACTCCCTTCAACGTGATAACGATTCTCTCCATGCAGAGATTAAACAGCTGAGAGCAAAACTTAACGTGGACGGTATCAGCAGAAGCAGTAGTAACGCGTCGACGGAAGAAAACGTCTTAGTAAAGGCGGATGAAATGGTGATGCCTAGTAACGAAGTCTTAGAGCTAAACCAGCGTCCTTTGCCACCGCCACCACATATTCCTACGGAAGCTCCGGCATTGGAGCTTGAATACGAGATGTTGAGCATTTTCCCACGTGCGGAAATCTTCAGAGAAGATCCTGCTGATAGTAGCGACTCAAGCGCTATTCTGAACGAGGATTATAGTCCCACGGCGGCTGAAGTGGCCGCGGCTACGGCAGTTGAAATGTCGACGATGGGATGTTTTGGCCAATTTGTGAAAATGGAAGAGCATGAAGATCTTTTTAGTGGAGAGGAAGCTTGCAAGTTGTTTGCAGATAATGAGCAGTGGTATTGTTGA

>BnaA01HB16

ATGATGAAAAGACTAAGCAGCTCAGATTCAATGTGTGGTCTAGTCTCCAATTCTCCAGATGAGCAGAGTCCACGAGGGTACGGAGGTAATTTCCAGTCTATGCTCGACGGCTACGAAGAAGACGGCACAATAGTCGAGGAATATTCCGGCAACCACCACCACATGGGCCTATCGGAGAAGAAGAGGAGGCTGCGTGTTGACCAAGTCAAAGCTCTCGAGAAGAATTTCGAGCTTGAGAACAAACTCGAGCCCGAGAGGAAAACCAAACTAGCGCAAGAGCTTGGTCTTCAGCCTCGCCAAGTAGCGGTTTGGTTCCAAAACCGCCGTGCACGGTGGAAGACAAAACAGCTCGAAAAAGACTACGGCCTTCTCAAGAGCCAGTACGACTCTCTCCGCCACAACTTCGACTCGCTCCGCCGCGACAACGATTCTCTTCTCCAAGAGATTAGTAAAATGAAAGGTAAGATCAACGGAGAAGAAGAAGATAACAACAACGTCAAGGCCACGACGGAGAGTGATATCTCCGCCGTGAAGGAAGAAGAAGATCCGATTCCTTCGTCTCCTCCTCAGTTTCTCGAACACTCGACCGGTTTTAACTACCGGCGAAGCTTCACCGATCTCCGTGATCTTATGCCCAACCCCGTCGTCGAAGCTGGATCTTCCGACAGCTGCGACTCGAGCGCTGTTCTAAACGAAGAAACCAGCTCGGAGAATGGGAGATTGACGCCGCCGGCGACGGTCGCCGGCGGGAGTTTCTTGCAGTTCGTGAAAACAGAGGATCACGACGAGTTTTTTAGCGGCGAAGAAGCTTGTGGCGGCTTCTTCTCCGACGTGCAACCGCCGTCTCTGAATTGGTACTCCGCGTCTGATCACTGGACATGA

>BnaC01HB16

ATGATGAAAAGACTAAGCAGCTCAGATTCAATGTGTGGTCTAGTCTCCAATTCTCCAGATGAGCAGAGTCCACGAGGGTACGGAGGTAATTTCCAGTCTATGCTCGACGGCTACGAAGAAGACGGCACAATAGTCGAGGAATATTCCGGCAACCACCACCACATGGGCCTATCGGAGAAGAAGAGGAGGCTGCGTGTTGACCAAGTCAAAGCTCTCGAGAAGAATTTCGAGCTTGAGAACAAACTCGAGCCCGAGAGGAAAACCAAACTAGCGCAAGAGCTTGGTCTTCAGCCTCGCCAAGTAGCGGTTTGGTTCCAAAACCGCCGTGCACGGTGGAAGACAAAACAGCTCGAAAAAGACTACGGCCTTCTCAAGAGCCAGTACGACTCTCTCCGCCACAACTTCGACTCGCTCCGCCGCGACAACGATTCTCTTCTCCAAGAGATTAGTAAAATGAAAGGTAAGATCAACGGAGAAGAAGAAGATAACAACAACGTCAAGGCCACGACGGAGAGTGATATCTCCGCCGTGAAGGAAGAAGAAGATCCGATTCCTTCGTCTCCTCCTCAGTTTCTCGAACACTCGACCGGTTTTAACTACCGGCGAAGCCTCACCAATCTCCGTGATCTTATGCCCAACCCCGTCGTCGAAGCTGGATCTTCCGACAGCTGCGACTCGAGCGCTGTTCTAAACGAAGAAACCAGCTCGGAGAATGGGAGATTGACGCCGCCGGCGACGGTCGCCGGCGGGAGTTTCTTGCAGTTCGTGAAAACAGAGGATCACGACGAGTTTTTTAGCGGCGAAGAAGCTTGTGGCGGCTTCTTCTCCGACGTGCAACCGCCGTCTCTGAATTGGTACTCCGCGTCTGATCACTGGACATGA

>BnaA08HB16

ATGAAGAGACTTAGCAACTCAGATTCAATGTGTGGTCTGATCTCCAATTCCACAGATGAGCAGAATCCACGAGGGTACGGGCATAATTTCCACTCTATGCTTAACGGTTACGAAGAAGACGGTACAACGGTCGAGGAATATTCCGGCAACCACCACATGGGTCAATCAGAGAAGAAGAGGAGGTTACGTGTTGACCAAGTCAAAGCTCTCGAGAAGAACTTCGAGCTCGAGAACAAAATCGAACCTGAGAGAAAAACACAACTAGCACAAGAGCTTGGACTTGAACCTCGTCAAGTAGCGGTTTGGTTTCAGAACCGCCGTGCACGGTGGAAGACAAAACAGCTCGAAAAAGATTACGGCCTTCTTAAGAGCCAGTACGACTCTCTCCGCCACAACTTCGACTCGCTCCGCCGCGATAACGACTCGCTTGTTTTAAAGATTAGTGAACTCAAAGCTAAGATCAACGGAGAAGACGTTAACAACGACAGCAAGGTTACGGCGGAGAGTGATATCTCCGCCGTGAAAGAAGAGAATGTTCCTTCGTCTCCTCCTGAGTTTATAGAACATTCCACCGGCTTTGACTACCGGCGAAGCTTCACCGATCTCTGTGACCTTCTACCGAACTCCACCGCCCCCGACGGTGGATCTTCCGACAGCTGCGATTCGAGCGCTGAAACCAGCTCCGAGAACGGAAGATTGACGCCGCCGCCGACGGTTACCGGCGGAAATTTTCTACAGTTCGTGAAAACAGAGCAGATGGAGGATCACGACGACTTTCTGAGCGGGGAAGAAGCGTGTTGTTTCTTCTCCGATGAGCAACCACCGTCTCTTCATTGGTACTCCGCCTCTGATCACTGA

>BnaC03HB16

ATGAAGAGACTTAGCAGCTCAGATTCAATGTATGGTCTGATCTCCAATTCCACAGATGAGCAGAGTTTTCGAGGGTACGGACATAATTTCCAGTCTATGCTTGATGGTTACGAAGACGACAGTACAATGATGGAGGAATACTCCGGCAACCACCACATGGGTCAATCGGAGAAGAAGAGGAGGTTACGTGTTGACCAAGTCAAAGCTCTCGAGAAGAACTTCGAGCTCGAGAACAAACTCGAACCTGAGAGGAAAACAAAACTAGCACAAGAGCTTGGACTTGAACCTCGTCAAGTAGCGGTTTGGTTTCAGAACCGCCGTGCACGGTGGAAGACAAAACAGCTGGAAAAAGATTACGGCCTTCTTAAGAGCCAGTACGACTCTCTCCGCCACAACTTCGACTCGCTCCGCCGCGATAACGACTCGCTTGTTTTAAAGATTAGTGAACTCAAAGCTAAGATCAACGGAGAAGAAGATAACAACAAGGTTACGGCGGAGAGTGATATCTCCGCCGTGAAAGAAGAGAATGTTCCTTCGTCTCCTCCTGAGTTTATAGAACATTCCACCGGCTTTGAGTACCGGCGAAGCTTCACCGATCTCTGTGACCTTCTACCCAACTCCACCGCTCCCGACGGTGGATCCTCCGACAGCTGCGATTCGAGCGCCGAAACCAGCTCCGAGAACGGAAGATTGACGACGCCGACGGTTACCGGCGGAAATTTATTTCAGTTCGTGAAAACAGAGCAGATGGAGGATCATGACGACTTTCTGAGCGGTGAAGAAGGGTGTTGTTTCATCTCCGATGAGCAACCACCGTCTCTTCATTGGTACTCCGCCTCTGATCACTGAACATGA
