# Supplementary material for: Functional and regulatory diversity of homeobox-leucine zipper transcription factors BnaHB6 under dehydration and salt stress in Brassica napus L
Source: Plant Mol Biol. 2024 May 15;114(3):59. doi: 10.1007/s11103-024-01465-6 (PMC11096223; doi:10.1007/s11103-024-01465-6)
Supplement: Supplementary file 3 — Supplementary file3 (DOCX 483 KB) [file 11103_2024_1465_MOESM3_ESM.docx]

**Figure S1**. Sequence comparison and structural characterization of *BnaHB6*s in *B. napus*. **A.** Sequence similarity between four *B. napus* homologues. **B.** Gene structure of four *BnaHB6* genes. The purple boxes and lines indicate exons and introns, respectively. **C.** Domain organization of the BnaHB6 proteins in *B. napus*. The purple and pink boxes correspond to HD and LZ, respectively. The number of domains at the N- and C-termini is consistent with Arce et al. (2011). Putative phosphorylation sites (Ser, Thr and Tyr) are indicated by black circles. The location of domains was visualized using the Illustrator for Biological Sequences (IBS) program (<http://ibs.biocuckoo.org/>). **D.** Alignments of the *B. napus* nucleotide and deduced amino acid sequences. Introns in the nucleotide sequences are colored gray. HD and LZ regions in the amino acid sequences of the BnaHB6 proteins represent the homeodomain and leucine zipper motif, respectively.

A.

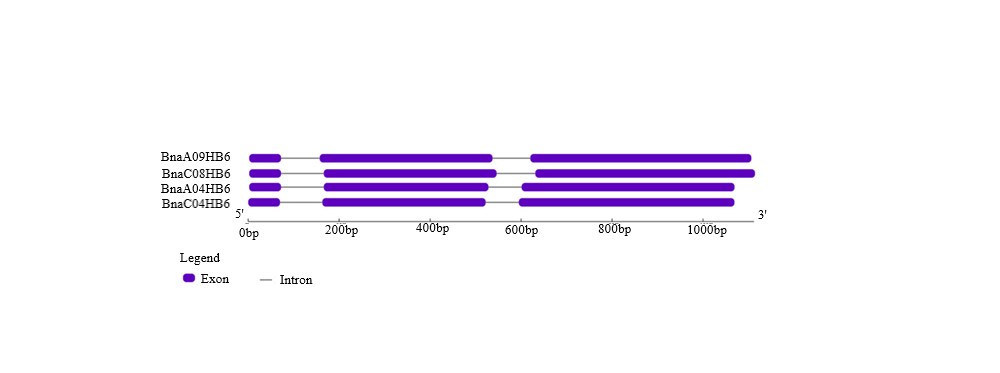
B.

C.


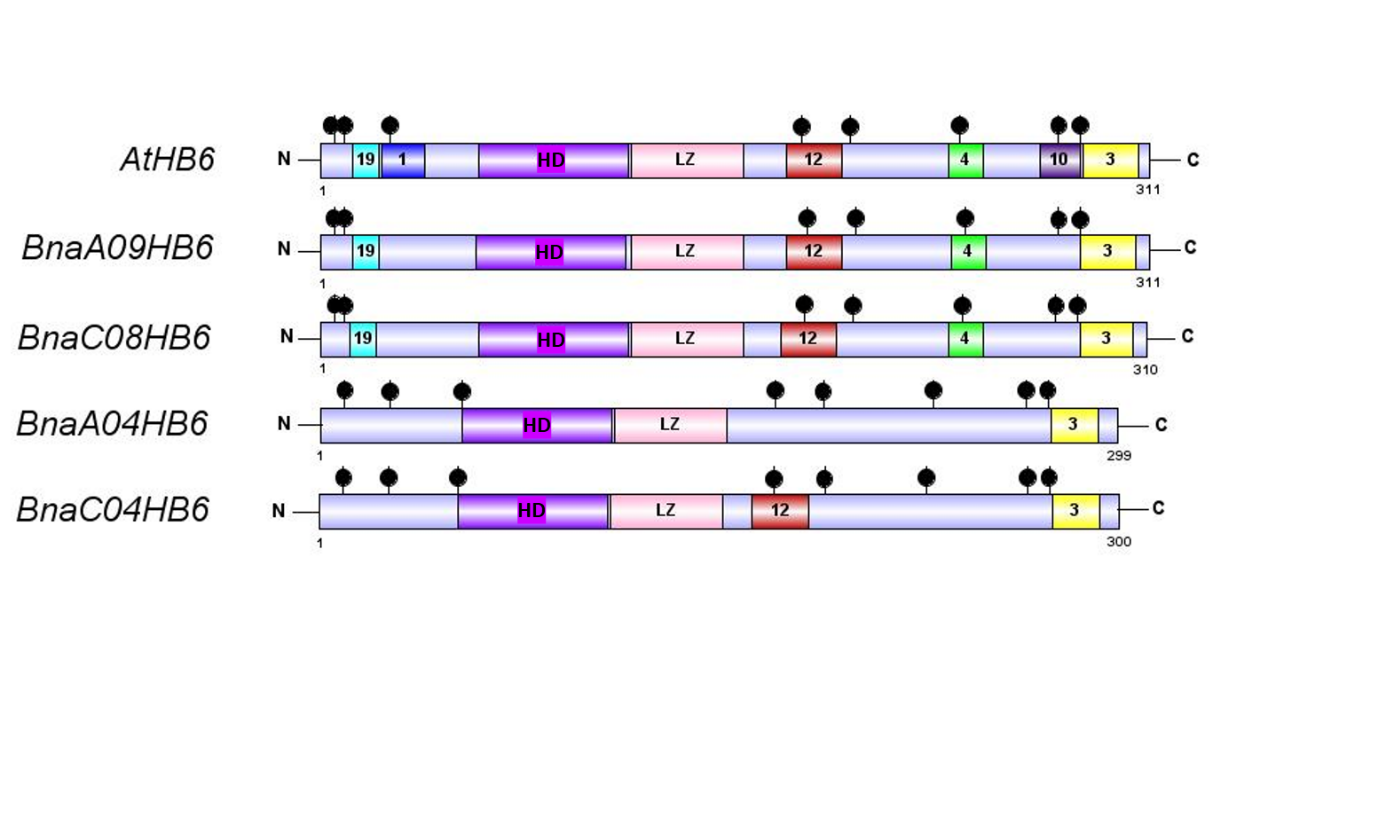


D.

BnaA09HB6 ATGATGAAGAGATTAAGCAGTTCAGATTCAGTGGGTGGTCTCATCTCTTTATGTCCCACT 60

BnaC08HB6 ATGATGAAGAGATTAAGCAGTTCAGATTCAGTGGGTGGTCTCATCTCTTTATGTCCCACT 60

BnaA04HB6 ATGATGAAGAGATTAAGAAGTTCAGATTCAGTGGGTGGTCTGATCTCTTTATGTCACACA 60

BnaC04HB6 ATGATGAAGAGATTAAGAAGTTCAGATTCAGTGGGTGGTCTGATCTCTTTATGTCACACA 60

***************** *********************** ************* ***

BnaA09HB6 ACTTCCACAGGTTATTTTCTTATACCAATGTTTTAAAGTTAATATTCAAACAAGTCCTTT 120

BnaC08HB6 ACTTCCACAGGTTCTTTTCTTATACCAATGTTTTAAAGTTAATTTTCAAACAAGCCCTTA 120

BnaA04HB6 TCTTCTACAGGTTCTTAATCTTAACCTTTTTATTTAGCTAATTTTCTACCAAGCTTTTAA 120

BnaC04HB6 TCTTCTACAGGTTGTTAATCTTAACCTTTTTATTTAGCTAATTTTCTAGCAAGCCCTTAA 120

**** ******* ** * *** * * ** * * * * * * * *

BnaA09HB6 AATTTCATAATTTTAACCT---------CAATTTTGGCAAACAGATCAGCCGAGTCCAAG 171

BnaC08HB6 AATATCATAATTTTAACCACAAATTCCTAAATTTTGGCAAACAGATCAGCCGAATCCAAG 180

BnaA04HB6 TTTCAATTTTGTTTACCTTAAAATTACCTCAATTCGTCAAACAGATGAGCAGAGTCCAAG 180

BnaC04HB6 TTTCATA-ATTTTTACCTTCAAATTACCTCAATTCGTCAAACAGATGAGCAGAGTCCAAG 179

* **** * * ** * ********* *** ** ******

BnaA09HB6 AAGATACGGGAGAGAGTTTCAGTCGATGCTTGAAGGTTACGAGGAGGAAGAAGAAGAAGC 231

BnaC08HB6 AAGATGCGGGAGAGAATTTCAGTCGATGCTCGAAGGTTACGAGGAGGAAGAAGAAGAAGC 240

BnaA04HB6 AAGATACGGGT------------CGATGCTTGAAGGTTACGATGAGGAAGAAGAAGAAGA 228

BnaC04HB6 AAGATACGGGT------------CGATGCTTGAAGGTTACGATGAGGATGAAG---AAGA 224

***** **** ******* *********** ***** **** ***

BnaA09HB6 CGTAACCGAGGAAAGAGGACAAACCGGTTTAGCCGAGAAGAAGAGACGGTTAAGCATTAA 291

BnaC08HB6 CATAACCGAGGAAAGAGGACAAACCGGTTTAGCCGAGAAGAAGAGACGGTTAAACATTAA 300

BnaA04HB6 AG------CGATAACCGAGGAGAGAGGACAAACGGAGAAGAAGAGACGGTTAAGCATTAA 282

BnaC04HB6 AG------CGATAGCTGAGGAGAGAGGACAAACTGAGAAGAAGAGACGGTTAAGCATTAA 278

* * * * * ** * * ******************* ******

BnaA09HB6 CCAAGTTAAAGCCTTGGAGAAAAATTTCGAGTTAGAGAACAAGCTTGAGCCCGAGAGGAA 351

BnaC08HB6 CCAAGTTAAAGCCTTGGAGAAAAATTTCGAGTTAGAGAACAAGCTTGAGCCTGAGAGGAA 360

BnaA04HB6 CCAAGTGAAAGCCTTGGAGAAGAACTTTGAGTTAGAGAACAAGCTTGAGCCTGAGAGGAA 342

BnaC04HB6 CCAAGTCAAAGCCCTGGAGAAGAACTTCGAGTTAGAGAACAAGCTTGAGCCTGAGAGGAA 338

****** ****** ******* ** ** *********************** ********

BnaA09HB6 AGTGAAGCTAGCTCAAGAACTTGGTCTCCAACCTCGTCAAGTAGCTGTTTGGTTTCAGAA 411

BnaC08HB6 AGTGAAGTTAGCTCAAGAACTTGGTCTCCAACCTCGTCAAGTAGCTGTTTGGTTTCAGAA 420

BnaA04HB6 AGTGAAGTTAGCTCAAGAACTTGGTCTTCAACCTCGTCAAGTAGCTGTTTGGTTCCAGAA 402

BnaC04HB6 AGTGAAGTTAGCTCAAGAACTTGGTCTTCAAACTCGTCAAGTAGCTGTTTGGTTCCAGAA 398

******* ******************* *** ********************** *****

BnaA09HB6 CCGCCGTGCGCGGTGGAAGACAAAACAGCTCGAGAAAGATTACGGTGTTCTCAAAACGCA 471

BnaC08HB6 CCGCCGTGCGCGGTGGAAGACAAAACAGCTTGAGAAAGATTACGGTGTTCTCAAAACGCA 480

BnaA04HB6 CCGCCGCGCACGGTGGAAGACAAAACAGCTTGAGAAAGATTACGGTGTTCTTAAAACACA 462

BnaC04HB6 CCGCCGTGCACGGTGGAAGACAAAACAGCTTGAGAAAGATTACGGTGTTCTTAAAACCCA 458

****** ** ******************** ******************** ***** **

BnaA09HB6 GTACGATTCTCTCCGCCATAACTTCGATTCCCTCCGCCGTGACAATGAATCTCTCCTTCA 531

BnaC08HB6 ATACGATTCTCTCCGCCATAACTTTGATTCCCTCCGCCGTGAAAATGAATCTCTTCTTCA 540

BnaA04HB6 GTACGATTCTCTCCGCCATAACTTCGATTCCCTCCGCCGTGACAATGAATCTCTTCTTCA 522

BnaC04HB6 GTACGATTCTCTCCGCCATAACTTTGATTCCCTCCGCAGTGACAATGAATCTCTTCTTCA 518

*********************** ************ **** *********** *****

BnaA09HB6 AGAGGTAACAAAAATTCAGACAAGGGTTATAAAGTTGAGATCTCCGGTTACAAAGTCTGA 591

BnaC08HB6 AGAGGTAACAAAATTCAGACAAGGGTTATTAAAGCTGAGATCTCCGGTCGCAAAGTCTGA 600

BnaA04HB6 AGAGGTAATAAAAATTCAACCTTTTTCTGCTTAAAGATTCAAACT------------TTA 570

BnaC04HB6 AGAGGTAACAAAAATTCAAACTTTTTCTGCTTAAAGATTGAAACT------------TTA 566

******** **** * * * * *

BnaA09HB6 GACTTTT--TTTGTTTTGTCGTGGTGCAGATCGGTAAACTAAAAGCTAAGCTAAACGGAG 649

BnaC08HB6 GACTTTTTTTTTGTTTTGTCTTGATGCAGATCGGTAAACTAAAAGCTAAGCTTAACGGAG 660

BnaA04HB6 TGTTGAAAGATTGAAACTTTTTGATGCAGATTAGTAAACTGAAGTCTAAGCTTAACGGAG 630

BnaC04HB6 TGTTGAAAGATTGAAACTTTTTCATGCAGATTAGTAAACTGAAGTCTAAGCTTAACGGAG 626

* *** * * ******* ******* ** ******* *******

BnaA09HB6 AAGAAGAAGTTGAAGAAGATGATGAAGATGAAGAGAACAACGCGGTGACGATGGAGTGTG 709

BnaC08HB6 AAGAAGAAGGAGATGA---TGTTGATGAAGAAGAGAACAACTTGGCGACGATGGAGAGTG 717

BnaA04HB6 AAGAAGAAG------------------AGGAAGAGAATAACGCCGTGACGATGGAGAGTG 672

BnaC04HB6 GAGAAGAAG---------------AAGAGGAAGAGAACAACGCCGTGGCGATGGAGAGTG 671

******** * ******** *** * * ******** ***

BnaA09HB6 ATGTTTCCGTCAAGGAAGAAGAAGTTTCGTTGCCGGAGGAGCTTACAGA---TCCGCCGT 766

BnaC08HB6 ATGTTTCCGTCAAGGAAGAAGAAGTTTCGTTGCCGGAGCAGATCACAGA---GCCGCCGT 774

BnaA04HB6 ATTTTTCCGTCAAGGAGGAAGAAGATTCGTTGCCGGAAAATATCACAGA---ACCGCCGT 729

BnaC04HB6 ATTTTTCCGTCAAGGAGGAAGAAGTTTCGCTGCCGGAAAATATCACAGAAGAACCGCCGT 731

** ************* ******* **** ******* * * ***** *******

BnaA09HB6 CTTCTCCTCCGCAGCTTCTAGAACATTCCGACAGTTTCAATTACCGGAGTTTCACCGACC 826

BnaC08HB6 CTTCTCCTCCGCAGCTTCTAGAGCATTCCGACAGTTTCAATTACCGGAGTTTCACCGACC 834

BnaA04HB6 CGTCTCCTCCAGAGCTTCTAGAACATTCGGATAGTTTCAATTACCGGAGTTTTACCGATC 789

BnaC04HB6 CGTCTCCTCCAGAGCTTCTAGAACATTCGGATAGTTTCAATTACCGGAGTTTTACCGATC 791

* ******** ********** ***** ** ******************** ***** *

BnaA09HB6 TCCGCGACCTTCTTCCGTTAAAGGCCGCGGCTTCCTCCGTCGCCGCCGCTGGATCGTCGG 886

BnaC08HB6 TCCGCGACCTTCTTCCGTTAAAGGCCGCGGCTTCCTCCGTCGCCGCCGCTGGATCGTCGG 894

BnaA04HB6 TCCGGGATCTTCTTCCATTAAAGGCTGCGGCTTCTTCCTTCGCCGCCGCTGGATCGTCGG 849

BnaC04HB6 TCCGGGATCTTCTTCCATTAAAGGCTGCGGCTTCTTCCTTCGCCGCTGCTGGATCGTCGG 851

**** ** ******** ******** ******** *** ******* *************

BnaA09HB6 ACAGTAGCGATTCGAGCGCCGTGTTGAACGAGGAAAGTAGCTCCAACGTTACGGCGGGTC 946

BnaC08HB6 ACAGTAGCGATTCGAGCGCCGTGTTGAACGAGGAAAGTAGCTCTAACGTTACGGCGGCTC 954

BnaA04HB6 ACAGCAGCGATTCGAGCGCCGTGATGAACGAGGAGAGTAGCTCTAACGTCACGGTGGCTC 909

BnaC04HB6 ACAGCGGCGACTCGAGCGCCGTGTTGAACGAGGAGAGTAGCTCCAACGTCACGGTAACTC 911

**** **** ************ ********** ******** ***** **** **

BnaA09HB6 CGGTGACTGTTCCCAGCGGTGGTTTCTTGCAGTTTGTGAAAATGGAGCAGACGGAGGATC 1006

BnaC08HB6 CGGCGACGGTTCCCGGCGGCAGTTTCTTGCAGTTTGTGAAAATGGAGCAGACGGAGGATC 1014

BnaA04HB6 CGACGGCGGTTCCCGGCGGTGGTTTCTTTCAGTTTGTGAAAATGGAGCAGACGGAGGATC 969

BnaC04HB6 CGACGGCGGTTCCCGGCGGTAGTTTCTTCCAGTTTGTGAAAATGGAGCAGACGGAGGATC 971

** * * ****** **** ******* *******************************

BnaA09HB6 ACGACGACTTTCTGAGCGGAGAAGAAGCGTGCGGGTTTTTCTCCGATGAGCAGCCACCGT 1066

BnaC08HB6 ACGACGACTTTCTGAGTGGAGAAGAAGCGTGCGGGTTTTTCTCCGATGAACAGCCACCGT 1074

BnaA04HB6 ACGACGACTTTCTGAGTGGAGAAGAAGCTTGCGGTTTTTTCTCCGATGAACAGCCGCCGT 1029

BnaC04HB6 ACGACGACTTTCTGAGTGGAGAAGAAGCTTGCGGTTTTTTCTCCGATGAACAGCCGCCGT 1031

**************** *********** ***** ************** ***** ****

BnaA09HB6 CTCTGCACTGGTATTCCACCGTTGATCAGTGGAACTGA 1104

BnaC08HB6 CTCTGCACTGGTATTCCACCGTTGATCAGTGGAACTGA 1112

BnaA04HB6 CTCTACACTGGTACTCCGCCGTTGATCACTGGACTTGA 1067

BnaC04HB6 CTCTACACTGGTACTCCGCCGTTGATCACTGGACTTGA 1069

**** ******** *** ********** **** ***

A09HB6 MMKRLSSSDSVGGLISLCPTTSTDQPSPRRYGREFQSMLEGYEEEEE-EAVTEERGQTGL 59

C08HB6 MMKRLSSSDSVGGLISLCPTTSTDQPNPRRYGREFQSMLEGYEEEEE-EAITEERGQTGL 59

A04HB6 MMKRLRSSDSVGGLISLCHTSSTDEQSPRRYG----SMLEGYDEEEEEEAITEERGQ--- 53

C04HB6 MMKRLRSSDSVGGLISLCHTSSTDEQSPRRYG----SMLEGYDEDE-EEAIAEERGQ--- 52

***** ************ *:***: .***** ******:*:* **::*****

**HD**

A09HB6 AEKKRRLSINQVKALEKNFELENKLEPERKVKLAQELGLQPRQVAVWFQNRRARWKTKQL 119

C08HB6 AEKKRRLNINQVKALEKNFELENKLEPERKVKLAQELGLQPRQVAVWFQNRRARWKTKQL 119

A04HB6 TEKKRRLSINQVKALEKNFELENKLEPERKVKLAQELGLQPRQVAVWFQNRRARWKTKQL 113

C04HB6 TEKKRRLSINQVKALEKNFELENKLEPERKVKLAQELGLQTRQVAVWFQNRRARWKTKQL 112

:******.******************************** *******************

**LZ**

A09HB6 EKDYGVLKTQYNSLRHNFDSLRRDNESLLQEIGKLKAKLNGEEEVEEDDEDEENNAVTME 179

C08HB6 EKDYGVLKTQYDSLRHNFDSLRRDNESLLQEIGKLKAKLNGEEEEEEDVDEEENNLATME 179

A04HB6 EKDYGVLKTQYDSLRHNFDSLRRDNESLLQEISKLKSKLNGEEEEE----EEENKAVKME 168

C04HB6 EKDYGVLKTQYDSLRHNFDSLRSDNESLLQEISKLKSKLNGGEEEE----EEENNAVAME 167

***********:********** *********.***:**** ** :***: . **

A09HB6 CDVSVKEEEVSLPEELT-DPPSSPPQLLEHSDSFNYRSFTDLRDLLPLKAAASSVAAAGS 238

C08HB6 SDVSVKEEEVSLPEQIT-EPPSSPPQLLEHSDSFNYRSFTDLRDLLPLKTAASSVAAAGS 238

A04HB6 SDFSVKEEEDSLPENIT-EPPSSPPELLEHSDSFNYRSFTDLRDLLPLKAAASSFAAAGS 227

C04HB6 SDFSVKEEEVSLPENITEEPPSSPPELLEHSDSFNYRSFTDLRDLLPLKAAASSFAAAGS 227

.*.****** ****::* :******:***********************:****.*****

A09HB6 SDSSDSSAVLNEESSSNVTAGPVTVPSGGFLQFVKMEQTEDHDDFLSGEEACGFFSDEQP 298

C08HB6 SDSSDSSAVLNEESSSNATAAPATVPGGSFLQFVKMEQTEDHDDFLSGEEACGFFSDEQP 298

A04HB6 SDSSDSSAVMNEESSSNVTVAPTTVPGRGFFQFVKMEQTEDHDDFLSGEEACGFFSDEQP 287

C04HB6 SDSGDSSAVLNEESSSNVTVTPTAVPGGSFFQFVKMEQTEDHDDFLSGEEACGFFSDEQP 287

***.*****:*******.*. *.:**. .*:*****************************

A09HB6 PSLHWYSTVDQWN 311

C08HB6 PSLHWYSTVDQWN 311

A04HB6 PSLHWYSAVDHWT 300

C04HB6 PSLHWYSAVDHWT 300

*******:**:*.
