# Supplementary material for: Functional and regulatory diversity of homeobox-leucine zipper transcription factors BnaHB6 under dehydration and salt stress in Brassica napus L
Source: Plant Mol Biol. 2024 May 15;114(3):59. doi: 10.1007/s11103-024-01465-6 (PMC11096223; doi:10.1007/s11103-024-01465-6)
Supplement: Supplementary file 4 — Supplementary file4 (DOCX 72 KB) [file 11103_2024_1465_MOESM4_ESM.docx]

Figure S2. BnaC08HB6 protein structure and location of point mutations in the *bnac08hb6* mutant. **A.** Schematic of the BnaC08HB6 protein structure with locations of point mutations predicted to affect protein function. The amino acid substitution corresponding to the nonsense mutation is indicated by an asterisk; **B.** Sequence alignment of the BnaC08HB6 protein in WT and the *bnaco8hb6* mutant with marked point mutations.


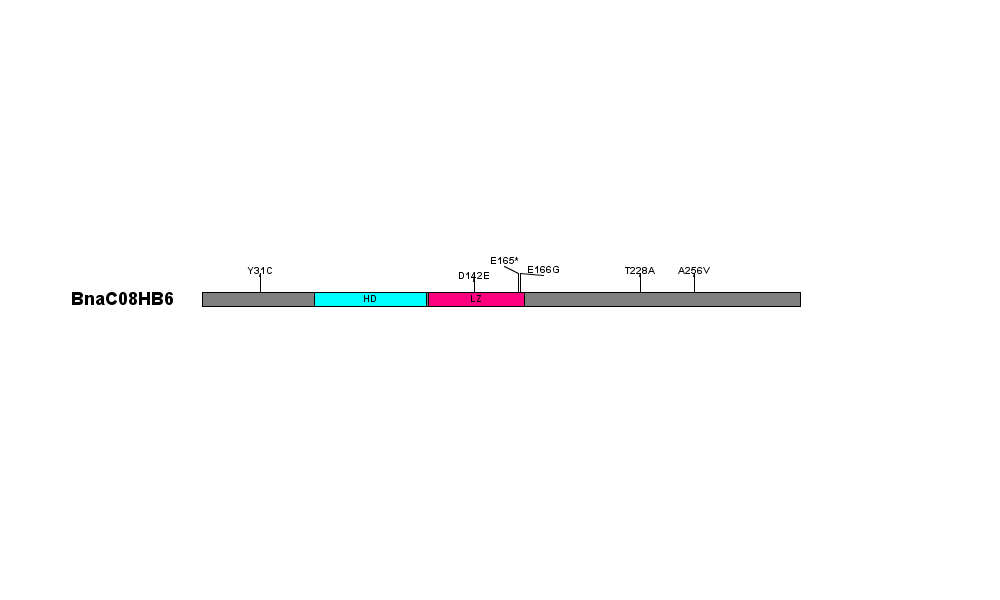
A.

B.

BnaC08HB6 MMKRLSSSDSVGGLISLCPTTSTDQPNPRRYGREFQSMLEGYEEEEEEAITEERGQTGLA 60

bnac08hb6 MMKRLSSSDSVGGLISLCPTTSTDQPNPRRCGREFQSMLEGYEEEEEEAITEERGQTGLA 60

****************************** *****************************

HD

BnaC08HB6 EKKRRLNINQVKALEKNFELENKLEPERKVKLAQELGLQPRQVAVWFQNRRARWKTKQLE 120

bnac08hb6 EKKRRLNINQVKALEKNFELENKLEPERKVKLAQELGLQPRQVAVWFQNRRARWKTKQLE 120

************************************************************

LZ

BnaC08HB6 KDYGVLKTQYDSLRHNFDSLRRDNESLLQEIGKLKAKLNGEEEEEEDVDEEENNLATMES 180

bnac08hb6 KDYGVLKTQYDSLRHNFDSLRRENESLLQEIGKLKAKLNGEEE-GDDVDEEENNLATMES 179

**********************:******************** :**************

BnaC08HB6 DVSVKEEEVSLPEQITEPPSSPPQLLEHSDSFNYRSFTDLRDLLPLKTAASSVAAAGSSD 240

bnac08hb6 DVSVKEEEVSLPEQITEPPSSPPQLLEHSDSFNYRSFTDLRDLLPLKAAASSVAAAGSSD 239

***********************************************:************

BnaC08HB6 SSDSSAVLNEESSSNATAAPATVPGGSFLQFVKMEQTEDHDDFLSGEEACGFFSDEQPPS 300

bnac08hb6 SSDSSAVLNEESSSNVTAAPATVPGGSFLHFVKMEQTEDHDDFLSGEEACGFFSDEQPPS 299

***************.*************:******************************

BnaC08HB6 LHWYSTVDQWN 311

bnac08hb6 LHWYSTVDQWN 310

***********

nonsense mutation

non-conservative missense mutation

conservative missense mutation
