# Supplementary material for: Functional and regulatory diversity of homeobox-leucine zipper transcription factors BnaHB6 under dehydration and salt stress in Brassica napus L
Source: Plant Mol Biol. 2024 May 15;114(3):59. doi: 10.1007/s11103-024-01465-6 (PMC11096223; doi:10.1007/s11103-024-01465-6)
Supplement: Supplementary file 5 — Supplementary file5 (PPTX 4243 KB) [file 11103_2024_1465_MOESM5_ESM.pptx]

## Slide 1
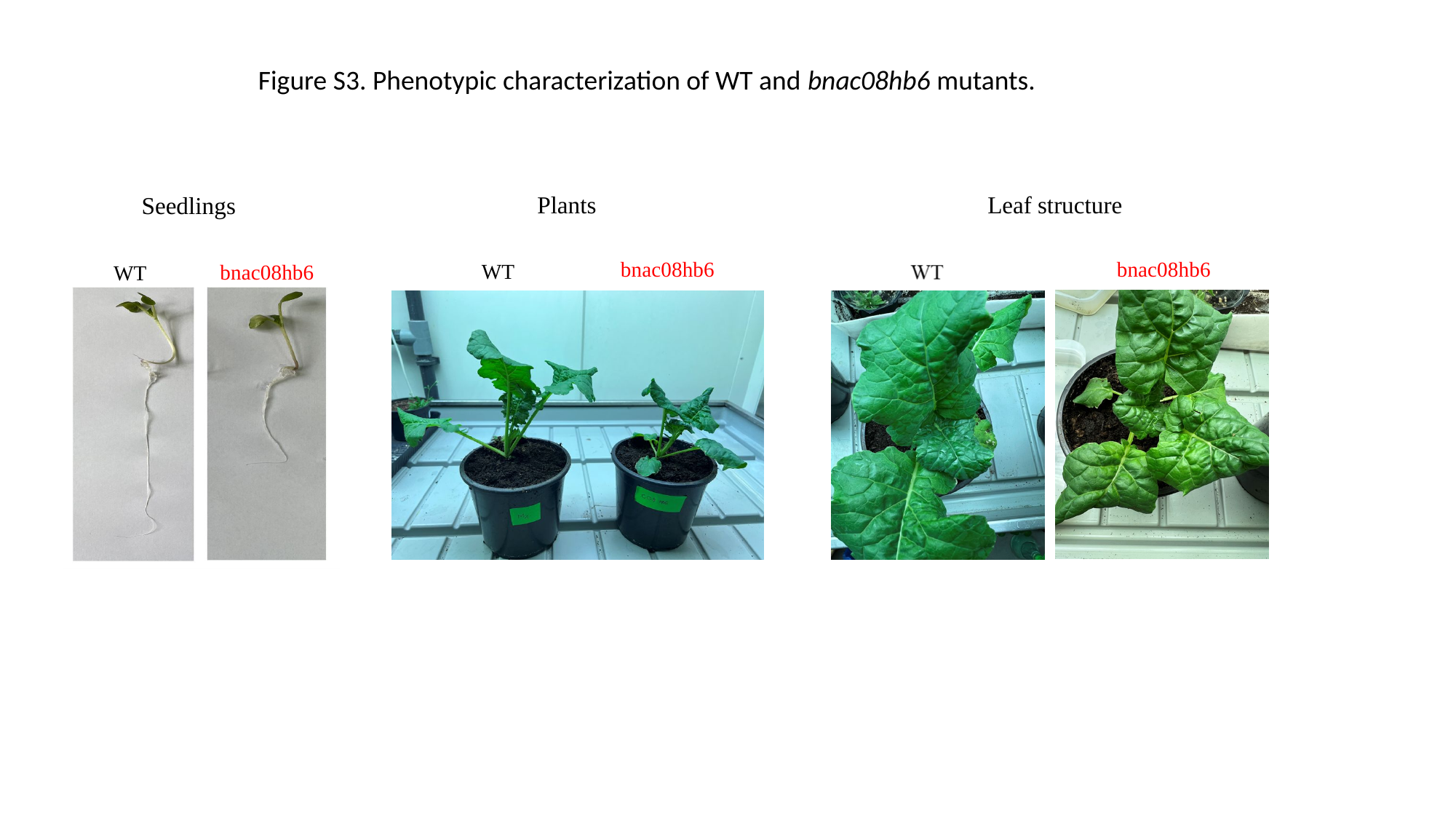

Figure S3. Phenotypic characterization of WT and bnac08hb6 mutants.
Plants
Leaf structure
Seedlings
bnac08hb6
bnac08hb6
WT
bnac08hb6
WT
