# Supplementary material for: Functional and regulatory diversity of homeobox-leucine zipper transcription factors BnaHB6 under dehydration and salt stress in Brassica napus L
Source: Plant Mol Biol. 2024 May 15;114(3):59. doi: 10.1007/s11103-024-01465-6 (PMC11096223; doi:10.1007/s11103-024-01465-6)
Supplement: Supplementary file 6 — Supplementary file6 (PPTX 1846 KB) [file 11103_2024_1465_MOESM6_ESM.pptx]

## Slide 1
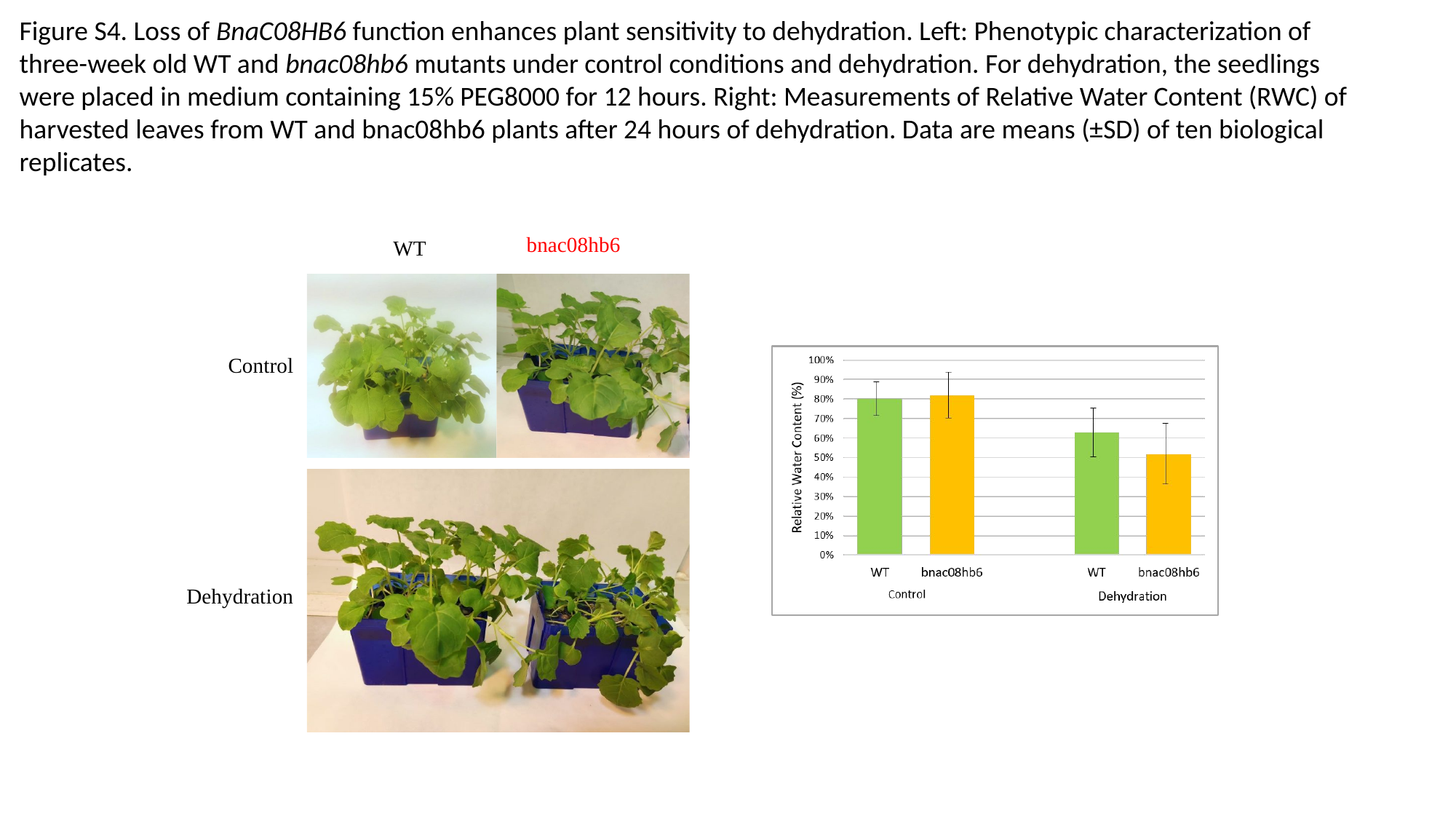

Figure S4. Loss of BnaC08HB6 function enhances plant sensitivity to dehydration. Left: Phenotypic characterization of three-week old WT and bnac08hb6 mutants under control conditions and dehydration. For dehydration, the seedlings were placed in medium containing 15% PEG8000 for 12 hours. Right: Measurements of Relative Water Content (RWC) of harvested leaves from WT and bnac08hb6 plants after 24 hours of dehydration. Data are means (±SD) of ten biological replicates.
bnac08hb6
WT
Control
Dehydration
